# An injectable hydrogel containing *N*-acetylglycine for the treatment of Gaucher disease

**DOI:** 10.1039/d5ra04610f

**Published:** 2025-10-28

**Authors:** Lipi Pradhan, Sumit Manna, Amar Jeet Yadav, Bajrang Bajrang, Debayani Chakraborty, Khushboo Bhagat, Shikha Tripathi, Avanish Singh Parmar, Aditya K. Padhi, Sudip Mukherjee

**Affiliations:** a School of Biomedical Engineering, IIT (BHU) Varanasi UP 221005 India sudip.bme@iitbhu.ac.in +91-7980659213; b Laboratory for Computational Biology & Biomolecular Design, School of Biochemical Engineering, IIT (BHU) Varanasi UP 221005 India aditya.bce@iitbhu.ac.in; c Department of Physics, IIT (BHU) Varanasi UP 221005 India

## Abstract

Gaucher disease (GD) is a rare inherited genetic disorder resulting from a recessive mutation in the GBA1 gene, which encodes the glucocerebrosidase (GCase) enzyme, resulting in the accumulation of glycolipids within the lysosomes of various body organs. Recently, small molecules that act as stabilizers for mutant proteins have become popular as a potential treatment for genetic disorders. Small-molecule stabilizers improve the stability of mutant proteins by facilitating proper folding, allowing them to regain their functional conformation. This prevents the proteins from entering the proteosomal degradation pathway, ultimately restoring their biological functions. Exploration of novel small-molecule stabilizers of GCase through rational process pipelines using *in vitro* and *in vivo* studies is quite expensive and time-consuming. A comprehensive process pipeline was developed for the pre-clinical identification of new GCase stabilizers using a stepwise funnel selection approach. Initially, small molecules were screened through a high-throughput molecular docking approach to repurpose these already existing molecules known for their protein stabilizing ability and evaluate their potential to stabilize mutant GCase, for the treatment of GD. Based on the intermolecular interactions and docking scores, the top two leads, *N*-acetylglycine (NAG) and deoxycholic acid (DCA), were further evaluated for their ability to stabilize the GCase enzyme through extensive molecular dynamics simulations and binding free energy analyses. Following detailed *in vitro* and *in vivo* toxicity studies, NAG was selected as the lead for further studies in *in vitro* and *in vivo* GD models. NAG increased the GCase activity in both chemically and siRNA-induced gene knockdown GD cell models, indicating NAG as a GCase stabilizer. The enhancement in GCase activity and improved motor behavior observed in the CBE-induced Gaucher mouse model following treatment with NAG confirmed its stabilizing efficacy. Finally, a single dose of NAG containing injectable hydrogel was developed and effectively used to treat GD and improve overall survivability.

## Abstract 2

Injectable hydrogel containing *N*-acetylglycine acts as GCase stabilizer.

## Graphical abstract

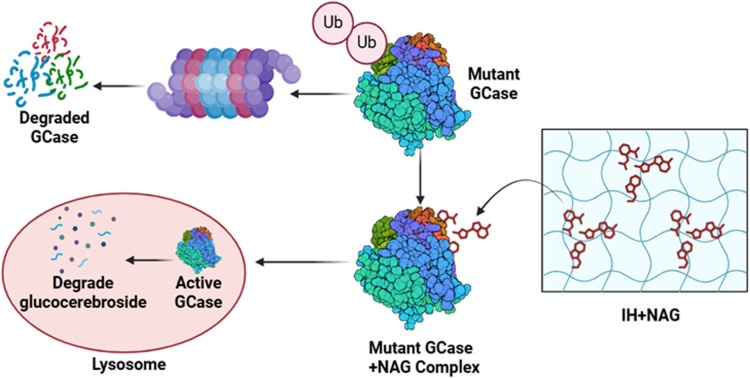

## 1. Introduction

GD is a widely known lysosomal storage disease occurring due to a biallelic mutation in the Glucosylceramidase Beta 1 (*GBA1*) gene affecting 1/50 000 to 1/100 000 people.^1^ A lysosomal hydrolase protein of 60 kDa molecular weight, called GCase (EC 3.2.1.45), is encoded by the *GBA1* gene.^2^ GCase is found in the lysosome of every cell containing the nucleus and is responsible for breaking the glycosidic bond in glucosylceramide, also known as glucocerebroside, and its derivatives, producing glucose and ceramide.^3^ Alteration of the *GBA1* gene results in mutant GCase, and deficiency of GCase fails to degrade glucocerebroside, causing accumulation of these substances in various organs and contributing to a multi-systemic rare autosomal recessive metabolic disorder known as GD.^4^ Mutations in the *GBA1* gene can alter the normal stability of the GCase enzyme, either provoking rapid degradation through the proteasome or a reduction in GCase activity, resulting in the accumulation of glucocerebroside, mainly affecting the bone marrow, liver, spleen, lymph nodes, central nervous system (CNS), lungs *etc.*^5,6^ Deposition of these glycolipid components leads to hepatosplenomegaly, suppressed hematopoiesis, cytopenia, bone lesions, interference with several cellular processes, triggers secondary inflammatory and immune reactions, and sometimes provokes ER stress response, causing the accumulation of insoluble synuclein aggregates in brain tissues, resulting in neuronal injury and degeneration as seen in neuronopathic forms of GD.^7,8^ Around 700 different variants of the *GBA1* gene mutation have been reported to date, highlighting the complex nature of GD.^9^ The primary hallmark of GD is a buildup of Gaucher cells, causing enlargement of organs, mainly the spleen and liver. Other symptoms include anemia, epistaxis, thrombocytopenia, delayed growth and puberty, bone disorders with acute and chronic pain, bruising, cognitive impairment, muscle weakness, *etc.*^1^ GD is classified into three broad categories based on the onset, severity, and neurological involvement.^10^ The most common non-neuronopathic form of GD is GD1 (Gaucher disease type 1), which can occur at any stage of life, and the symptoms can vary from patient to patient. GD2 (Gaucher disease type 2) and GD3 (Gaucher disease type 3) are neuropathic forms.^11^ Currently, enzyme replacement (ERT), substrate reduction therapy (SRT), and supportive care are practiced for the treatment of GD.^10^ Although these strategies benefit non-neuropathic types, they fail to be effective for the neuropathic forms. Additionally, these therapies are time-consuming, costly, and invasive, requiring continuous administration and causing discomfort and side effects.^1^ As a result, the treatment and management of GD demand the development of novel strategies to treat both neuropathic and non-neuropathic forms of GD.

Small molecules have gained interest in treating rare genetic disorders in the last few decades. These are low-mass chemical compounds having a molecular weight of less than 500 Da that are derived from natural or synthetic molecules.^12,13^ These small molecules can enter the cells, bind to the mutant protein, stabilize it against denaturation, and help achieve its functionality.^14^ In addition, they prevent ER-associated degradation systems by binding to the protein and promoting proper folding, allowing the protein to reach its target site. Additionally, they can cross the blood–brain barrier (BBB), effectively addressing neurological symptoms of the disease. These small molecule stabilizers can binds to the mutant enzymes as a reversible inhibitor and help them properly translocate to their targeted site, where they are replaced by the natural substrate in higher concentration.^15,16^ Previous reports demonstrated potential effects of small molecules across a range of protein misfolding diseases, including Pompe disease, Krabbe disease, hemophilia A, Huntington's disease, *etc.*^12,17,18^

Various small molecules, including ambroxol, isofagomine, NCGC758, NCGC607, *N*-acetylcysteine is explored for the treatment of GD.^19–23^ Although these molecules have been found to be beneficial in improving GCase activity, they require continuous administration, which increases the overall treatment cost and risk of inflammation and side effects. In our study, we have developed a single-administrative hydrogel formulation containing a small-molecule stabilizer that can maintain GCase activity for a longer duration.

A thorough literature study was performed to select 30 existing small molecules known for their stabilizing activity that are studied in various other genetic disorders. However, they had not been previously studied for their stabilizing effects in GD. The objective of this study was to repurpose these already known small molecules and evaluate their potential to stabilize both wild-type and mutant forms of GCase, for potential treatment of GD.

**Fig. 1:**
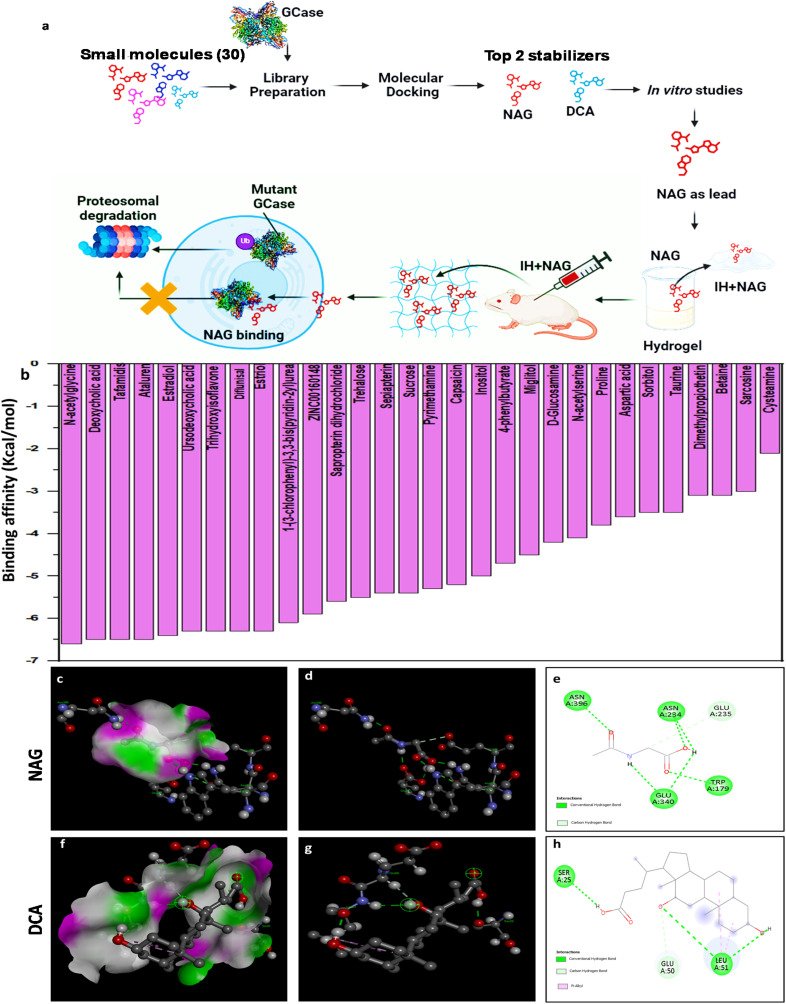
Molecular docking analysis for screening of lead small molecules: (a) schematic illustration showing a screening of small molecules utilizing a molecular docking approach and evaluation of the GCase stabilizing ability of the identified small molecule in the CBE-induced GD mouse model. Graph showing (b) binding affinity of all the screened small molecules with wild-type GCase, (c–h) 3D and 2D images of molecular interaction between NAG and DCA with wild-type GCase.

Virtual screening of small molecules with the ability to target GCase is a time-saving and cost-effective strategy that can quickly identify a promising, ready-to-use protein stabilizer. In our present research, we performed drug repurposing to screen 30 different small molecules using a computational molecular docking approach to identify the top two small molecules that can stabilize the mutant GCase and enhance its activity. The top identified leads, NAG and DCA, underwent biocompatibility studies *in vitro* and *in vivo* to examine their toxicity effects on biological systems. Based on the scores of interactions and biocompatibility, NAG was identified as the top lead. The molecular docking and simulation studies of NAG with wild-type GCase and mutant GCase revealed that NAG can stabilize the protein, even outperforming the stability observed in wild-type conditions. The role of NAG as a GCase stabilizer was confirmed experimentally using chemically induced and siRNA-based gene knockdown *in vitro* cell models. The potential of NAG as a GCase modulator was further investigated in the CBE-induced Gaucher mouse model. The promising results of NAG promoted the development of an injectable hydrogel formulation containing NAG (referred to as IH + NAG) for sustained NAG release, aiming to provide a single-administered treatment approach for long-term treatment of GD, thereby reducing the overall cost of the therapy and burden of multiple injections. One single injection of IH + NAG could retain GCase function, maintain motor ability, and improve the survivability of the rodents tested in the Conduritol B Epoxide (CBE) induced Gaucher mouse model (Fig. 1a).

## 2. Methods and materials

### 2.1. Materials

Recombinant human-derived GBA/Glucosylceramidase (rGCase) was obtained from MedChemExpress. SASI_Mm02_00313113 Predesigned siRNA was obtained from Sigma-Aldrich. Dulbecco's Modified Eagle's Medium (DMEM) and Opti-MEM were procured from Himedia. PBS was brought from Gibco. 4-Methylumbelliferyl-β-glucopyranoside (4MU-β-Glc) was brought from TCI (Tokyo Chemical Industry). 3-(4,5-Dimethylthiazol-2-yl)-2,5-diphenyltetrazolium bromide (MTT), NAG, DCA, DPPH (2,2-diphenyl-1-picrylhydrazyl), Triton X-100, sodium taurocholate, citric acid, sodium phosphate dibasic, glycine, high and low-viscous sodium alginate, calcium chloride, Bradford reagent, and xylene were procured from SRL (Sisco Research Laboratories Pvt. Ltd). RIPA buffer was obtained from Thermo Scientific. CBE was obtained from BLD Pharma. 4′,6-Diamidino-2-phenylindole (DAPI), primary antibody (GBA Polyclonal Antibody, PA5-21347), secondary antibody (Goat anti-Rabbit IgG, Alexa Fluor™ 594, A-11037), and Lipofectamine were brought from Thermo Fisher Scientific. Raw 264.7 (mouse macrophagic cell line) and HEK 293 (human embryonic kidney cell line) were purchased from the National Centre for Cell Science (NCCS), Pune, India.

### 2.2. Molecular docking analysis for screening of GCase stabilizers

A virtual screening using a molecular docking strategy was performed to identify the top 2 GCase stabilizers. We selected the 30 small molecules based on their previously reported protein stabilizing ability in various diseases.^23,24^ However, they had not been previously studied for their stabilizing potential in GD. Structure-based screening was performed using a fast AutoDock-based molecular docking approach in CB-Dock2 to evaluate the binding affinity and interaction between the selected small molecule and GCase. The three-dimensional (3D) structure of wild-type monomeric human β-glucocerebrosidase (WT-GCase) was taken from the Protein Data Bank (PDB). The 3D structure of all the screened small molecules was obtained in the structural data file (SDF) format from the PubChem database.

### 2.3. Cytocompatibility and biocompatibility of the top two leads

The cell viability assay and chorioallantoic membrane (CAM) assay were performed to assess the cytocompatibility and biocompatibility of NAG and DCA. The detailed experimental procedure is provided in the SI.

### 2.4. GCase activity assay in CBE-induced Raw 264.7 cells

The effects of the top two identified GCase stabilizers in improving the GCase activity were assessed *in vitro* in CBE-induced GD cell models as per published literature with slight modification.^25^ Initially, 10 000 Raw 264.7 cells were seeded in each well of 96-well plates supplemented with DMEM media. The cells were allowed to grow for 24 hours. Following the incubation period, the media were replaced with fresh media. Various concentrations of NAG and DCA, starting from 5 μM to 100 μM, were mixed in the wells. In order to inhibit the GCase activity of cells, 0.5 mM CBE was added to each well and kept for another 24 hours. Untreated cells served as controls. The following day, the cells were rinsed using PBS and incubated with 8 μL of 0.2 M acetate buffer and 8 μL of PBS (pH 4.0). The reaction was initiated by mixing 10 μL of 5 mM 4-MUG, followed by incubation at 37 °C for 1 hour. 200 μL of 0.2 M glycine buffer (pH 10.7) was added to terminate the reaction, and the liberated 4-MU was measured using a multiple reader.^26^

### 2.5. GCase inhibition assay

It has been reported that small molecules at sub-inhibitory concentrations can bind to the targeted protein as competitive inhibitors and assist in achieving the proper conformation of the mutant enzyme, thereby allowing the accurate translocation of the mutant protein to the lysosome. Once the mutant-enzyme-inhibitor complex reaches the lysosome, the inhibitor, at sub-inhibitory concentrations, can be replaced by highly accumulated glucocerebroside, allowing the enzyme to function effectively.^27,28^ We performed a GCase inhibition assay to determine the concentration of NAG required to inhibit 50% of GCase activity under *in vitro* conditions. Initially, 15 ng of rGCase was incubated with nearly 3 mM of 4MU-β-Glc and various concentrations of NAG in McIlvaine buffer (0.2 M phosphate buffer, 0.1 M citrate, at pH 5.2) supplemented with 0.25% sodium taurocholate and 0.1% Triton X-100 (100 μL final volume) for 30 minutes at 37 °C. 0.4 M NaOH and 0.4 M glycine were added to the solutions at an equal volume, and fluorescence release from 4-methylumbelliferone (4MU) was measured (excitation 355 nm, emission 460 nm) using a multi-plate reader. The IC50 (half-maximal inhibitory concentration) value of NAG was calculated by fitting the graph with a growth/sigmoid function using Origin software.^29^

### 2.6. Transfection of HEK293 cells with predesigned siRNA for the knockdown of the *GBA1* gene

The role of NAG in enhancing the GCase activity was further evaluated in the siRNA-induced Gaucher cell model. Predesigned siRNA for GBA1 (SASI_Mm02_00313113 Predesigned siRNA, cat. no. PDSIRNA2D) was transfected in HEK293 cells line using the manufacturer's protocol with slight modifications. A master-mix solution was prepared by diluting 5 pmol of siRNA duplexes in 50 μL of Opti-MEM media. 50 μL of this freshly prepared master mix was poured into per well of a 96-well plate. 1.25 μL of Lipofectamine was then added into the well. The plate was then placed on a rocker shaker for 5 minutes, followed by incubation for 15 minutes. 7500 HEK293 cells in 125 μL of complete medium (without antibiotics) were then added to each well and homogenized by rocking the plate. Some wells were treated with 25 μM NAG and 25 μM NAC to study the effects of NAG in GBA knockdown cell models. The plate was then incubated for 72 hours at 37 °C. After the incubation period, the media was removed and the cells were incubated with 200 μL of 1% Triton X for 15 minutes. The GCase activity of these cell lysates was measured.

### 2.7. Effects of NAG in siRNA-induced Gaucher mouse model

The role of NAG as a potent stabilizer of GCase was further evaluated in siRNA-induced GD mouse model. Initially, 3 μg of siRNA (SASI_Mm02_00313113) was diluted in 50 μL of Opti-MEM. 2 μL of Lipofectamine 2000 reagent was added to the diluted siRNA and incubated for 15 minutes at room temperature. After the incubation period, this siRNA master mix was injected directly into the IP region of the mouse to develop the siRNA-induced gene knockdown Gaucher mouse.^30^ Balb/c mice (weight = 13–20 grams, age = 4–6 weeks) were randomly assigned to 4 experimental groups: group 1: received 3 μg of siRNA (denoted as siRNA; *n* = 3); group 2: received 5 μg of siRNA along with simultaneous injection of NAG (150 mg per kg body weight; *n* = 3); group 3: received 3 μg of siRNA along with simultaneous injection of NAC (150 mg per kg body weight; *n* = 3), group 4: healthy mice denoted as control (without siRNA; *n* = 3). After 72 hours post-siRNA injection, the mice were sacrificed, and their organs were collected and weighed. The brain, liver, and spleen tissues were homogenized and analyzed for GCase activity percent in treated and untreated groups.

### 2.8. Molecular docking and simulation of NAG with mutant and wild-type GCase

Molecular docking studies were performed to determine how NAG binds with the mutant and wild-type GCase enzyme to stabilize the mutant structure and maintain proper binding. The interaction of NAG with both mutant and wild-type GCase was further assessed employing molecular docking and simulation in a dynamic time dependent condition.

#### 2.8.1. Protein and ligand structure procurement and preprocessing

The protein and ligand structures were procured for molecular docking and simulation studies. WT-GCase and GCase-N370S (mutant-GCase) enzyme crystal structures were retrieved from the PDB with PDB IDs: 8AWR and 3KEH, respectively. The 3D structure of the NAG was obtained in the structural data file (SDF) format from the PubChem database. The structure of NAG was converted from the SDF to mol2 format using PyMOL. Water molecules, ligands, and other heteroatoms bound to the enzyme were removed before further processing. The pre-processed structures, including the WT-GCase, mutant-GCase, and NAG, were prepared and utilized for molecular docking studies. The details of molecular docking analysis of WT and mutant GCase with NAG, analysis of molecular interactions in GCase–NAG docked complexes, physicochemical properties, and free energy landscape from AAMD simulation trajectories, end-state binding free energy calculations for GCase–NAG Complexes are provided in the SI.

#### 2.8.2. Analysis of structural dynamics features in GCase–NAG complexes using all-atom molecular dynamics simulations

To investigate the stability, dynamics, and structural compactness of WT and mutant GCase–NAG docked complexes and the apo forms of GCase, we performed all-atom molecular dynamics (AAMD) simulations. These simulations using the GROMACS v.2022 package and CHARMM36 force field included four systems: WT-GCase, mutant-GCase, WT-GCase–NAG, and mutant-GCase–NAG docked complexes.^31,32^ Crystal structures of WT and mutant GCase (apo form) were controls for comparing the stability and structural dynamics of the docked complexes. System preparation was automated using a Python script leveraging Chimera's DockPrep tool to clean protein structures and incorporate missing residues using the Dunbrack rotamer library. Hydrogen atoms were explicitly added to NAG with custom force field parameters. The NAG coordinates (.gro), and topology (.itp) files were generated using the LigParGen and integrated with GCase files *via* the Python script.^33^ Each system was solvated in a cubic box using the SPCE water model, with a minimum 1.0 nm buffer from the box edges. Neutralization was achieved by adding Cl^−^ ions: two for WT-GCase, one for mutant-GCase, two for WT-GCase–NAG, and one for mutant-GCase–NAG. Energy minimization was conducted with the steepest descent algorithm for 50 000 steps under periodic boundary conditions. Equilibration was performed in canonical (*NVT*) and isobaric–isothermal (*NPT*) ensembles, followed by a 100 ns production run for each system under the *NPT* ensemble. Temperature and pressure were regulated using the Berendsen thermostat and Parrinello–Rahman barostat, respectively.^34,35^ A fs integration timestep was used, with a 1.2 nm cutoff for Coulomb interactions and a Fourier grid spacing of 0.16 nm. Long-range electrostatics were quantified using the Particle Mesh Ewald (PME) method, and bond lengths were constrained with the LINCS algorithm.^36,37^ This approach enabled a comprehensive evaluation of the structural integrity and interaction dynamics of GCase–NAG complexes under WT and mutant conditions.

#### 2.8.3. Mechanistic insights and interaction profiling of GCase–NAG complexes from AAMD simulations

To unravel the binding mechanisms, strength, and conformational dynamics of WT and mutant GCase complexes with NAG during AAMD simulations, equilibrated and stable trajectories (.gro and .xtc files) were analyzed using Visual Molecular Dynamics (VMD). Further analysis of stable simulation frames was conducted with GetContacts (https://getcontacts.github.io/), enabling precise identification and characterization of molecular interactions such as hydrophobic contacts, hydrogen bonds, and van der Waals forces. This comprehensive interaction profiling provided a detailed understanding of the binding dynamics and molecular interactions of both complexes. The detailed steps of molecular docking and simulation studies are provided in the SI file.

### 2.9. Synthesis of IH + NAG

To develop a single-administered IH + NAG, sodium alginate, a natural polymer known for its biocompatibility, was utilized. Initially, a 2% (w/v) high and low-viscosity sodium alginate solution was prepared by dissolving the polymer in deionized water under stirring.^38^ After complete dissolution, a mixture of 60% low viscous and 40% high viscous sodium alginate solution was prepared. The resultant solution was filtered using a syringe filter to remove the impurities. NAG was added to the final solution at 1.8 g% (w/v) under stirring to prepare the injectable NAG–alginate mixture. A 0.5% CaCl_2_ solution was added to the NAG-containing alginate mixture, forming the *in situ* injectable hydrogel (IH + NAG), which was utilized directly on the animal in the subsequent studies. The experimental methods of stability and NAG release from hydrogel is provided in SI manuscript.

### 2.10. Characterization of IH + NAG

The properties of the synthesized IH + NAG were studied employing various characterization techniques. The refractive index of NAG and IH + NAG was studied using refractometers (Anton Paar). For the rheology of IH + NAG, 1 mL of IH + NAG was crosslinked with an equal volume of crosslinker. A hydrogel patch without NAG was kept for comparison. The rheological properties of these patches were studied using an Anton Paar MCR 72 Rheometer. The patches were then placed between the parallel plate geometry, with a 50 mm diameter and a 1 mm gap. A constant frequency of 10 rad s^−1^ was applied to examine the linear viscoelastic range (LVER). Moreover, to determine the storage modulus, *G*′, and the loss modulus, *G*′′, a frequency sweep was performed. The apparent viscosity of IH + NAG was evaluated across a shear rate range of 1 to 100 s^−1^. To determine the gelation kinetics of IH + NAG, oscillatory time sweeps were conducted at a 10% radial strain and a constant frequency of 1 Hz.^39^

### 2.11. *In vivo* stabilizing activity of NAG in CBE-induced GD model

The *in vivo* stabilizing activity of NAG toward GCase enzyme was studied using motor behavior and GCase assay of the CBE-induced Gaucher mouse model. The *in vivo* studies in mice were conducted after the IAEC approval (IIT(BHU)/IAEC/2023/II/076; approval date: Aug 25, 2023). The mice were kept in polystyrene cages, maintaining hygienic conditions and easy access to water and food.

#### 2.11.1. Gaucher mouse model

It has been reported that CBE, an inhibitor of GCase at a concentration of 50 mg per kg per day decreases GCase activity by 68–88% in BALB/c micedoi.^40^ Balb/c mice of post-natal day 8 at a body weight of 3–4 g were selected and randomly divided into 3 groups: group 1: intraperitoneal (I.P) injections of 25 mg kg^−1^ day^−1^ of CBE for 10 days (denoted as CBE; *n* = 3); group 2: intraperitoneal (I.P) injections with CBE (25 mg kg^−1^ day^−1^ for 10 days; *n* = 3) along with simultaneous treatment of NAG (30 mg kg^−1^ day^−1^ for 10 days); denoted as (CBE + NAG; *n* = 3); group 3: untreated mice as control (without CBE; *n* = 3). Weight was measured every alternate day.

#### 2.11.2. Motor behavior

Continuous CBE administration into the mouse model has the ability to inhibit GCase function, resulting in the accumulation of harmful glucocerebroside inside various organs, including the brain. Deposition of these substrates hinders normal motor functions, causing limb impairment and difficulty in movement.^40^

The motor behavior of all the groups was evaluated using a wire-hanging test. Each mouse was placed on a hanging wire, and based on the duration they could hold the wire, they were given a score. The hang wire test was repeated 3 times for each mouse with a 1 minute interval between each repetition. The conditions and scores are summarized in Table S1.^41^

#### 2.11.3. *In vivo* stabilizing activity of IH + NAG in CBE-induced GD model

The stabilizing property of IH + NAG was studied in GD mouse model. The 15 day-old Balb/c mice with body weight of 5–6 g were randomly divided into 4 groups: group 1: intraperitoneal (I.P) injections of 50 mg kg^−1^ day^−1^ of CBE for 10 days (denoted as CBE); group 2: intraperitoneal (I.P) injections with CBE (50 mg kg^−1^ day^−1^ for 10 days) along with simultaneous subcutaneous treatment of NAG (150 mg kg^−1^ day^−1^ of single dose; *n* = 3); denoted as (NAG; *n* = 3); group 3: intraperitoneal (I.P) injections with CBE (50 mg kg^−1^ day^−1^ for 10 days) along with subcutaneous administration of injectable hydrogel containing NAG (150 mg kg^−1^ w.r.t. NAG for single dose; denoted as IH + NAG; *n* = 3) along with equal volume of CaCl_2_; group 4: untreated mice as control (without CBE; *n* = 3). Weight was measured every alternate day. The motor activity of experimental groups was evaluated using a wire-hanging test. Following 12 days of treatment, the mice were sacrificed, and the organs were collected to measure the GCase activity. A separate set of mice (*n* = 3) was kept for the survival study for the first three groups.

#### 2.11.4. GCase activity assay using artificial substrate

The effects on IH + NAG in enhancing the GCase activity at various organs were measured using the GCase activity assay. After 10 doses, all the experimental group animals were sacrificed, followed by the isolation of the brain, liver, and spleen to conduct GCase activity assay. The organs were cut into smaller pieces and homogenized using RIPA buffer. The insoluble solution was then centrifuged for 10 minutes at 17 000 g, maintaining 4 °C. The supernatant was collected, and the protein was estimated using the Bradford assay. 50 μg of protein from each sample was collected and added to 100 μL of working solution (5 mM 4MU-β-Glu, pH ∼ 5.5) and incubated for 1 hour at 37 °C covered from light. The reaction was then terminated by adding 200 μL of stop solution buffer (0.5 M glycine, pH approx. 10). The fluorescence intensity was measured using a multi-plate reader (excitation 355 nm and emission 460 nm).^21,42^ The following formula calculates the GCase activity:GCase activity (%) = fluorescence intensity of sample/fluorescence intensity of control × 100

### 2.12. Immunostaining

Immunostaining of treated and untreated brain, liver, and spleen tissue samples was conducted to study the presence of GCase enzyme. The slides containing different tissue samples embedded in paraffin were deparaffinized by placing the slides in a hot air oven at 60 °C for 15 minutes. Then, the slides were kept in a xylene bath for 10 minutes and rehydrated using increasing ethanol concentrations. The slides were then rinsed using Milli-Q water and washed using the citric buffer. The slides were then incubated in a hot air oven for 5 minutes, maintaining the temperature at 100 °C, followed by 2 PBS washes. The slides were then rinsed utilizing Triton X (0.5%) and kept for 5 minutes at room temperature for drying, followed by PBS wash. The tissue sections were then blocked using a blocking solution containing BSA (0.6%) and Triton X-100 (0.3%) and incubated for 1 hour. The primary antibody (GBA Polyclonal Antibody) at 1 μg mL^−1^ concentration was added to each tissue sample and incubated overnight at 4 °C. The next day, the samples were washed twice using PBS and incubated with DAPI (15 μg mL^−1^) and secondary antibody (Goat anti-Rabbit IgG) at a 10 μg mL^−1^ concentration for 1 hour. Both primary (1 : 500) and secondary antibodies (1 : 200) were diluted using 0.3% BSA in 0.1% Triton X-100 before adding to the tissue samples. The immunostained slides were then observed under a confocal microscope (Zeiss, Model no. LSM-780), and the captured images were further processed with the help of ImageJ software.

### 2.13. Statistical analysis

All *in vitro* and *in vivo* experiments were conducted with at least three replicates to ensure accuracy and reliability. The data from each experiment were presented as mean values along with standard error. A one-way ANOVA was performed using Origin software to assess the statistical significance of the observed data.

## 3. Results and discussion

### 3.1. Molecular docking analysis identified NAG and DCA as top stabilizers, showing the highest binding affinity towards GCase

Different molecules were screened using a molecular docking approach to identify the role of these small molecules as GCase stabilizers that show the maximum binding affinity with the GCase. It has been reported that the higher the negative value of the binding energy of a molecule interacting with its target protein, the greater the affinity of the compound to form a stable complex with it.^43^ The molecular docking results demonstrate the binding affinity of all 30 small molecules following the interaction with the GCase protein (Fig. 1b). From the initial screening of docking results, the top two small molecules (NAG and DCA) were selected based on their highest binding affinity with GCase for further analysis.

Discovery Studio analyzer was employed to derive the 2D and 3D images of the molecular interactions between the small molecules and WT-GCase. This analysis examines the amino acid residues of the GCase that interact with the small molecule and the nature of the intermolecular interaction stabilizing the GCase–NAG complex. Fig. 1c and f display the aromatic structure of the GCase–drug complexes, namely with NAG and DCA, respectively, in 3D space along with the location of H-bonds. In the H-bonded regions, the donor molecules are represented in violet, whereas the acceptor region is shown in green. The 3-D images of the docked complexes indicate the interaction between amino acid residues of the GCase protein with the lead small molecules (Fig. 1d and g). The 2D interaction analysis further illustrates the nature of the bonds (represented by dotted lines) formed between the interacting amino acid residues and the small molecule (Fig. 1e and h).

NAG was found to interact with GCase at five different residues by forming six H-bonds with four amino acid residues, including Asn396, Asn234, Trp179, Glu340, and one C–H interaction at Glu235. DCA interacts with GCase at three distinct residues, forming three H-bonds with Ser25 and Leu51 and one C–H interaction at Glu50. Additionally, DCA binds to GCase at Leu51 by forming two pi–alkyl interactions. The docking findings revealed various types of interactions between lead stabilizers and GCase, including H-bonds, C–H bonds, and pi–alkyl bonds, which contribute to the stabilization of GCase.

Finally, we conducted molecular docking studies of *N*-acetylcysteine (NAC), a well-known stabilizer of GCase, and compared the results with those of NAG. Our analysis revealed that the NAC–GCase complex exhibited a slightly lower binding affinity (−5.1 kcal mol^−1^) and weaker molecular interactions, whereas the NAG–GCase complex showed a stronger binding affinity (−6.6 kcal mol^−1^) and more stable interactions (Fig. S1). These findings suggest that NAG has a greater potential to stabilize the enzyme, which may contribute to enhanced therapeutic efficacy.

### 3.2. *In vitro* and *in ovo* biocompatibility studies demonstrated that NAG is non-toxic and safe for biomedical applications

The biocompatibility of the lead small molecules was thoroughly examined through both *in vitro* and *in ovo* studies to determine their safety and effectiveness. An *in vitro* cell viability assay in HEK293 cell lines demonstrates that NAG is cytocompatible, even at a higher dose of 5 mM, indicating its relative safety and effectiveness.

**Fig. 2:**
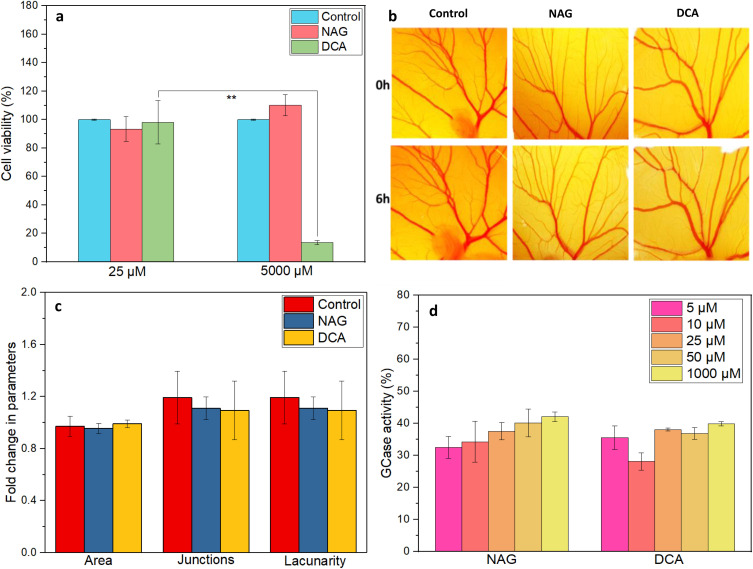
(a–c) Biocompatibility studies of the top 2 identified leads (a) cell viability assay of NAG and DCA at different concentrations, CAM assay: (b) microscopic images of developing blood vessels captured using a stereomicroscope at 0 and 6 h. (c) The graph shows fold change in different parameters of blood vessels exposed to NAG and DCA. (d) Graph showing GCase activity assay of lead stabilizers (NAG and DCA) in CBE induced Gaucher cell (Raw 264.7) model.

However, at a 5 mM dose, DCA exhibited a drastic reduction of cell viability (Fig. 2a), confirming its probable toxic effects in human cell lines.

The biocompatibility of NAG and DCA was further evaluated using the CAM (chorioallantoic membrane) assay on developing chicken embryos, which are particularly sensitive indicators of biocompatibility. The embryos exposed to NAG and DCA at a 5 mM dose demonstrate similar growth and vascular development compared to the control (untreated) chicken embryos till 6 hours (Fig. 2b). The quantification data showed that the fold change in area, junctions, and lacunarity of developing blood vessels is similar in the NAG and DCA-treated groups as that of the control embryos (Fig. 2c).

The cell viability assay is a direct toxicity assessment test in which cells are exposed to a toxic substance, resulting in direct effects. DCA at higher concentrations was found to cause harmful effects, reducing cell viability in its presence. Whereas an *in ovo* model is a complex three-dimensional model containing a functional circulatory system, including heart and blood vessels. DCA may show direct cytotoxic effects on individual cells, but the CAM's larger, integrated system can tolerate these effects without showing overall toxicity to the embryo or its vascular network.

### 3.3. NAG treatment in CBE-induced Raw 264.7 cells preserves the GCase enzyme activity

RAW 264.7 cells were exposed to CBE, an inhibitor of GCase, to develop a Gaucher cell model with reduced enzymatic activity. Both the identified lead GCase stabilizers (NAG and DCA) were then added at various concentrations to the cells to observe improvement in GCase activity. Both molecules exhibited a notable increase in GCase activity in a dose-dependent manner, suggesting a possible role in enhancing the enzymatic activity of GCase in CBE-induced Raw 264.7 cells (Fig. 2d). This enhancement in GCase level indicates the lead small molecule stabilizers have the ability to restore enzymatic activity and can help in treating GD. Based on the combined outcomes of the binding affinity, biocompatibility studies, and *in vitro* GCase activity assays in the CBE-induced GD model, NAG was identified as the lead GCase stabilizer and further studies were conducted using NAG.

### 3.4. GCase inhibition assay showed a decrease in the activity of human-derived rGCase in the presence of NAG

**Fig. 3:**
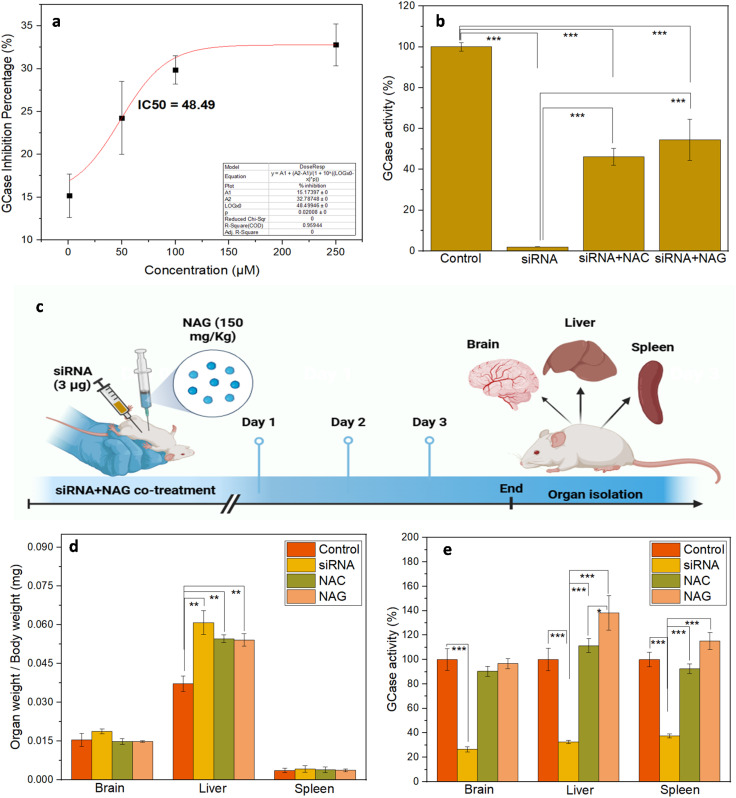
(a) The inhibition curve of NAG with rGCase determines the IC50 using the GCase-inhibition assay, (b) NAG enhances the GCase activity of siRNA-induced gene knockdown in HEK cell model *in vitro* (c) scheme showing treatment of siRNA induced knockdown GD mice with NAG, (d) organ weight of the in siRNA-induced GD mice exposed to NAG was found to be similar to the healthy control, (e) NAG enhanced the GCase activity in the brain, liver and spleen of the treated group (*n* = 3; *p < 0.05).

It has been reported that small-molecule stabilizers typically act as reversible antagonists or competitive inhibitors of targeted proteins, resulting in decreased activity. However, their primary aim is to stabilize intrinsically active yet unstable proteins, thereby preventing their clearance by the quality control system and facilitating their transport to the desired target location. Once the enzyme reaches its target, the small molecule is removed due to the presence of a high concentration of substrate, which competes with the small molecule and effectively restores the protein's activity.^31^ To verify that the selected lead small molecule binds to GCase effectively, a GCase inhibition assay using human-derived rGCase was performed. The results indicate a decrease in the activity of rGCase in the presence of NAG, indicating its probable binding to the active or allosteric sites of rGCase, thus inhibiting its function (Fig. 3a) with an IC50 value of 48.49 μM.

### 3.5. NAG enhances the GCase activity in siRNA-induced gene knockdown GD cell model

To confirm the GCase-stabilizing ability of NAG, HEK293 cell lines were transfected with predesigned siRNA targeting GBA1, causing gene knockdown and reduced GCase production. Small interfering RNA (siRNA) are widely used to induce RNA interference, that can inhibit messenger RNA (mRNA) that encodes the targeted protein in both *in vitro* and *in vivo* settings.^44,45^ A widely studied small molecule for the treatment of GD, NAG, was kept as a positive control.^46,47^ As expected, a significant reduction in GCase activity was observed in cells transfected with siRNA compared to untreated cells (Fig. 3b). On the other hand, the transfected cells incubated with NAG showed a nearly 54% enhancement in GCase activity, even more than the GCase activity observed in NAC (46%), further indicating the role of NAG as a stabilizer, which helps the enzyme reach its targeted site to perform its activity.

### 3.6. NAG enhances the GCase activity and prevents glucocerebroside accumulation inside organs of siRNA-induced gene knockdown GD cell model

The effects of NAG were further evaluated in the siRNA-induced GD mouse model. The mouse exposed to siRNA alone demonstrated an increase in organ weight, indicating the accumulation of glucocerebroside inside the brain, liver, and spleen due to siRNA-based knockdown of GCase mRNA, which led to GCase deficiency. The mice co-treated with NAC and NAG exhibited organ weights similar to those of healthy controls (Fig. 3c), suggesting that these two chaperones may be capable of stabilizing and trafficking the residual enzyme to its targeted site, thereby enhancing its activity and restoring the GCase activity of the residual protein in the brain and spleen. Additionally, a decrease in liver weight was observed in the groups exposed to NAG and NAC when compared to the untreated siRNA group. Moreover, siRNA administration reduced GCase activity in the brains, livers, and spleens of the untreated group. The groups co-treated with NAG showed enhancement in GCase activity in all the organs, outperforming the positive control, NAC (Fig. 3d).

### 3.7. Molecular docking and simulation of NAG with mutant and wild-type GCase

#### 3.7.1. Analysis of interaction strengths and binding affinities of GCase with NAG

**Fig. 4:**
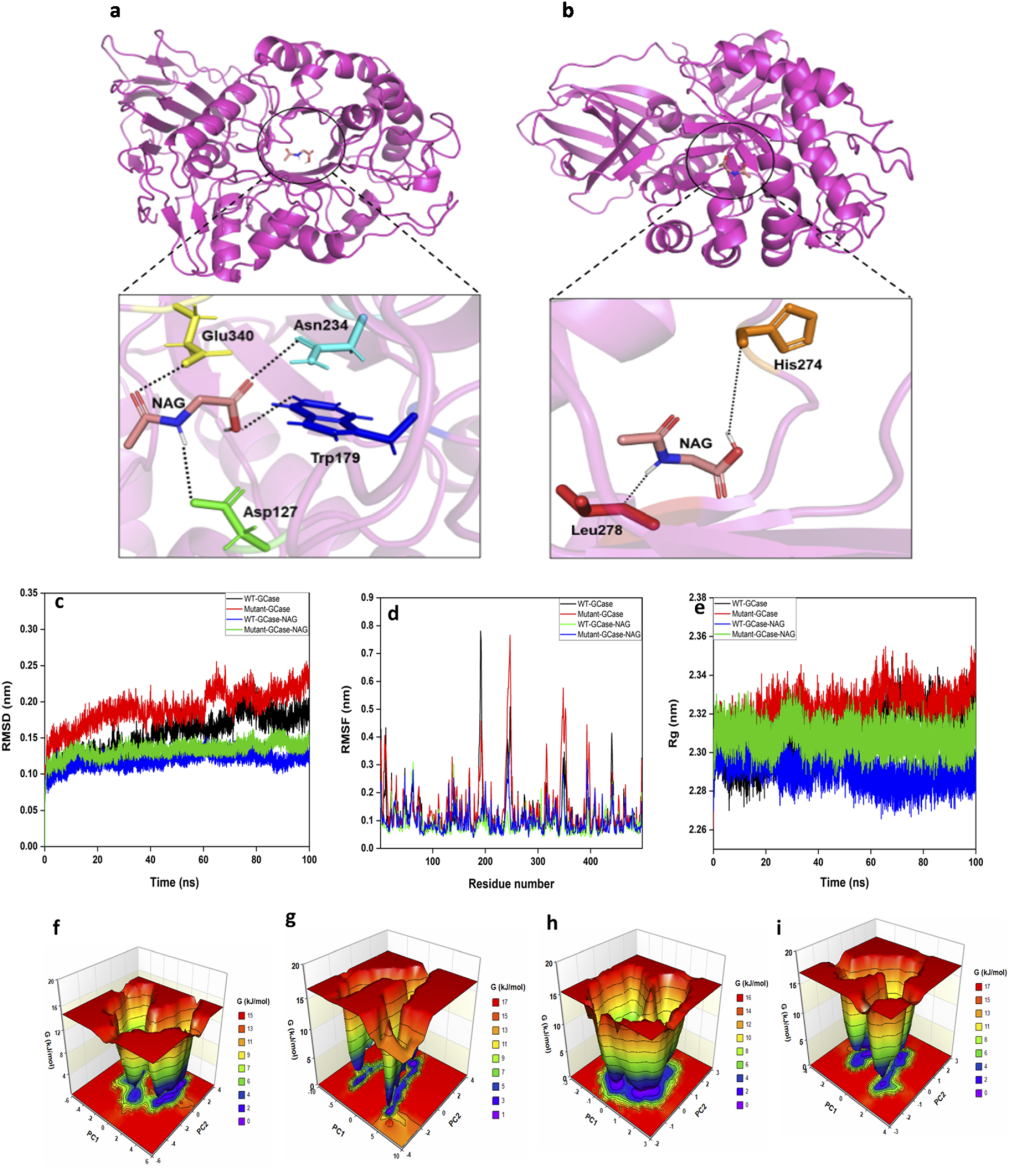
3D visualization of the docked complex of human GCase and its mutant bound with NAG. (a) Illustrates the interaction between WT-GCase and NAG. (b) Depicts the interaction between mutant-GCase and NAG. In all the figures interacting residues are represented in stick representation, and polar contacts are shown as black dashed lines. The structure–function-dynamic features and Gibbs free energy landscapes (FEL) of WT and mutant GCase and GCase–NAG complexes. The figure highlights the (c) backbone RMSD profiles for WT-GCase, mutant-GCase, WT-GCase–NAG, and mutant-GCase–NAG complexes; (d) RMSF profiles calculated for backbone atomic positions; and (e) *R*_g_ of Cα atoms for WT-GCase, mutant-GCase, WT-GCase–NAG, and mutant-GCase–NAG complexes throughout the AAMD simulation. The profiles of the WT-GCase, mutant-GCase, WT-GCase–NAG, and mutant-GCase–NAG models are depicted in black, red, blue, and green color, respectively. FEL contour plot for the, (f) WT-GCase, (g) mutant-GCase, (h) WT-GCase–NAG complex, and (i) mutant-GCase–NAG complex. In the FEL plots, color coding indicates energy states: red signifies high-energy states, yellow and green represent low-energy states, and blue and purple denote the most stable and lowest-energy states.

A blind docking study was performed to comprehensively explore potential binding sites of the selected small molecule on the enzyme surface. This was particularly important, as the exact binding modes of these molecules have not been previously established for this system. Molecular docking revealed distinct differences in binding affinities and interaction patterns between WT and mutant GCase with NAG. Notably, NAG binds to the active site of WT-GCase, whereas in the mutant GCase, our structural analysis revealed that NAG binds at a site distant from the active site, indicating a potential allosteric interaction. The WT-GCase demonstrated the highest binding affinity to NAG at −6.6 kcal mol^−1^, signifying robust and stable interactions (Fig. 4a). In contrast, the mutant-GCase–NAG complex showed a reduced binding affinity of −4.2 kcal mol^−1^, indicating weakened interactions within the binding pocket due to mutation-induced structural and conformational changes (Fig. 3b). In the WT-GCase–NAG complex, key residues such as Asn234, Asp127, Glu340, and Trp179 formed stabilizing hydrogen bonds with NAG. Conversely, the WT complex formed more hydrogen bonds and hydrophobic interactions than the mutant, reinforcing its superior binding affinity (Fig. 4b).

Detailed views of the active site region highlight these structural differences, with WT-GCase residues (magenta, blue) and their corresponding mutant residues (green, red) shown in (Fig. S2). Structural superimposition illustrates that the N370S mutation induces significant conformational and orientational shifts, disrupting the native binding pocket. In WT-GCase, NAG binding is stabilized by well-defined hydrogen bonds, while in the mutant form, these changes alter NAG's binding position within the active site. Conversely, the mutant complex displayed altered hydrogen bonding, involving His274 and Leu278.

#### 3.7.2. Analysis of structure-dynamics-stability and physicochemical properties of GCase and GCase–NAG models using AAMD simulations

AAMD simulations, performed over 100 ns for four systems (WT-GCase, mutant-GCase, WT-GCase–NAG, and mutant-GCase–NAG complexes), revealed critical insights into the structural stability, conformational dynamics, and physicochemical properties of these models (Table S2). RMSD analysis demonstrated distinct stability trends, with the WT-GCase–NAG complex displaying the highest stability, characterized by minimal fluctuations and an average RMSD of 0.11 nm. This was followed by the mutant-GCase–NAG complex (0.13 nm), WT-GCase (0.15 nm), and mutant-GCase (0.19 nm), which showed the greatest deviation and lowest stability (Fig. 4c). Per-residue RMSF profiles were examined to assess structural flexibility. The WT-GCase–NAG model exhibited the lowest flexibility (average RMSF: 0.08 nm), followed by the mutant-GCase–NAG complex (0.10 nm), WT-GCase (0.12 nm), and mutant-GCase (0.14 nm). These results underscore the relatively stable and restrained fluctuations in the WT-GCase–NAG system compared to the others (Fig. 4d).

The radius of gyration (*R*_g_) was calculated throughout the simulations to evaluate compactness. The WT-GCase–NAG model demonstrated the greatest compactness, with an average *R*_g_ of 2.29 nm, followed by the mutant-GCase–NAG complex (2.30 nm), WT-GCase (2.30 nm), and mutant-GCase (2.32 nm) having the least compactness (Fig. 4e). The RMSD, RMSF, and *R*_g_ analyses reveal that the WT-GCase–NAG and mutant-GCase–NAG complexes maintain greater structural stability and compactness compared to WT-GCase and mutant-GCase. The results also highlight the increased flexibility and instability of the mutant-GCase system, while the WT-GCase–NAG model demonstrated superior stability and compactness.

Principal component analysis (PCA) and free energy landscape profiling analysis confirmed stable PCA clusters for WT-GCase–NAG, mutant-GCase–NAG, WT-GCase, and mutant-GCase by evaluating the backbone atoms during equilibrated phases of the simulations. The compactness of these complexes was further confirmed through trace values of covariance matrices, with WT-GCase–NAG and mutant-GCase–NAG exhibiting values of 7.60 and 9.12 nm^2^, respectively, compared to 18.47 nm^2^ and 29.42 nm^2^ for WT-GCase and mutant-GCase. Gibbs free energy landscapes (FEL) generated from the first two principal components (PC1 and PC2) highlighted that the WT-GCase–NAG complex adopts more stable and compact conformations compared to its mutant counterpart and the unbound GCase models. This stability is evident in the FEL plots, where WT-GCase–NAG features more extensive deep blue low-energy basins, signifying stable low-energy states. At the same time, the mutant-GCase–NAG and other systems exhibit higher energy states (indicated by red regions) (Fig. 4f–i).

Additional binding free energy analysis of GCase–NAG complex using the MM/PBSA method revealed that the WT-GCase–NAG model exhibited the highest binding free energy of −19.01 kcal mol^−1^, significantly outperforming the mutant-GCase–NAG models, which showed binding affinities of −12.42 kcal mol^−1^ (Table S3). Furthermore, the binding energies of the complexes confirmed that the WT-GCase–NAG complex was more stable, with a total energy of −32494.3 kcal mol^−1^, compared to −32339 kcal mol^−1^ for the mutant complex (Table S4). These findings underscore the superior binding affinity and structural stability of the WT-GCase–NAG complex relative to its mutant counterparts.

#### 3.7.3. Interaction dynamics and intermolecular contacts in GCase–NAG complexes

**Fig. 5:**
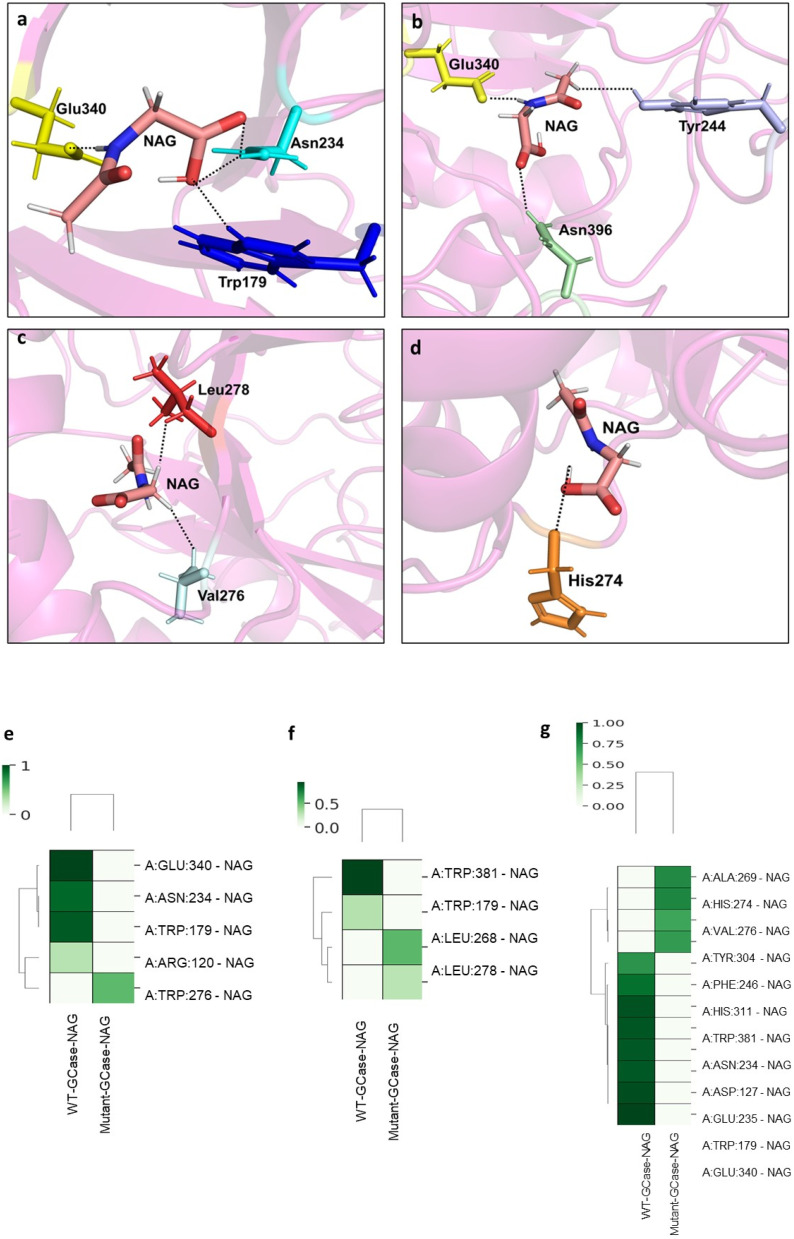
Mechanistic insights and molecular interactions in the WT and mutant GCase–NAG models at different time points of AAMD simulations. Panel (a) depicts the interacting residues of WT-GCase with NAG at the start of the simulation. Panel (b) illustrates the interacting residues of WT-GCase with NAG during the stabilized AAMD simulation. Panel (c) shows the interaction of NAG with the mutant-GCase residues at the start of the simulation. Panel (d) highlights the interaction of NAG with mutant-GCase residues during the stable course of AAMD simulation. In all the figures interacting residues are represented in stick format, and polar contacts are shown with black dashed lines. Comparative analysis of inter-molecular interactions in WT and mutant GCase–NAG complexes. Panel (e) depicts the hydrogen bond interactions, panel (f) shows the hydrophobic interactions, and panel (g) represents the van der Waals interactions, derived from equilibrated and stable time frames of AAMD simulation trajectories. In all contact maps, chain ‘A’ represents human GCase (receptor), while NAG (*N*-acetylglycine) represents the ligand molecule. The frequency of interactions is depicted by a gradient from green (high-frequency) to white (low-frequency). Figures (e–g) demonstrate that the WT-GCase–NAG complex forms more interactions than the mutant-GCase–NAG complex.

The trajectories were comprehensively analyzed to elucidate the structural dynamics, binding modes, and interaction strengths of WT and mutant GCase–NAG complexes during the AAMD simulations. At the onset of the simulation, the WT-GCase–NAG complex exhibited stable interactions, including hydrogen bonds with NAG mediated by residues Trp179, Asn234, and Glu340, a hydrophobic interaction with Trp381, and a salt bridge with His311 (Fig. 5a). These interactions were largely preserved throughout the simulation, with key residues such as Tyr244, Glu340, and Asn396 playing critical roles in stabilizing the complex (Fig. 5b). In contrast, the mutant-GCase–NAG complex displayed weaker initial interactions, forming a hydrogen bond involving residues Val276 and Leu278 and hydrophobic interactions with Leu278 (Fig. 5c). During the simulations, the mutant complex maintained a hydrogen bond with His274 and hydrophobic contacts involving Val230, Leu268, and Leu278 (Fig. 5d), indicating reduced binding strength and stability compared to the WT complex. Intermolecular contact maps were developed for each system to identify residues critical for stability and binding affinity, visualizing the frequency of hydrogen bonds, hydrophobic interactions, and van der Waals forces. The WT-GCase–NAG complex demonstrated higher contact frequencies at key residues, reflecting more stable and stronger interactions.

Notably, enhanced van der Waals interactions were observed at residue pairs A:PHE:246-NAG, A:HIS:311-NAG, A:TRP:381-NAG, A:ASN:234-NAG, A:ASP:127-B:NAG, A:GLU:235-NAG, A:TRP:179-NAG, and A:GLU:340-NAG (Fig. 5g). Frequent hydrogen bonds were detected at A:GLU:340-NAG, A:ASN:234-NAG, and A:TRP:179-NAG (Fig. 5e), while hydrophobic interactions primarily involved A:TRP:381-NAG and A:TRP:179-NAG (Fig. 5f). These findings highlight the critical role of van der Waals forces, hydrophobic interactions, and hydrogen bonding in strengthening GCase–NAG binding, significantly contributing to the stability and affinity of the WT complex across all interactions.

### 3.8. NAG increases the GCase activity in the CBE-induced Gaucher mouse model

**Fig. 6:**
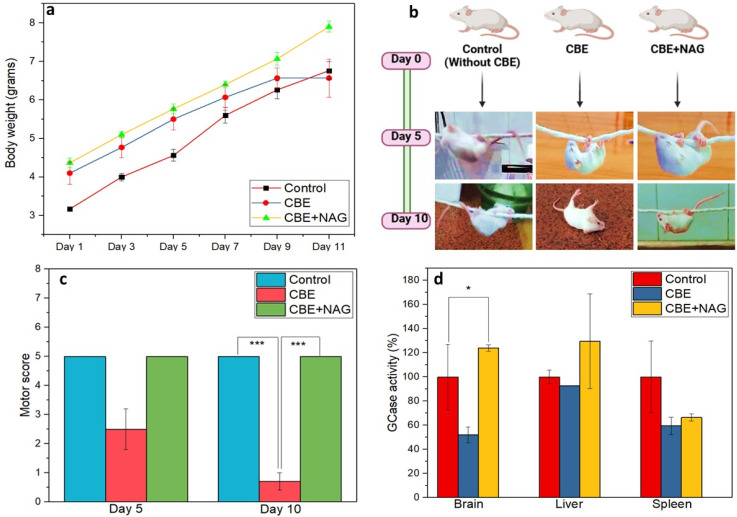
The protective effects of NAG in CBE-induced Gaucher mouse model (age-post-natal day 8): (a) graph showing the weight of the experimental group over time, (b) wire hanging test, (c) motor score obtained by experimental groups during wire hanging test, (d) GCase activity of the untreated and treated group after 10 days of CBE injection. A significant difference in motor score and GCase activity was observed in NAG-treated and untreated groups (*n* = 3; *p < 0.05).

The effects of NAG were studied in CBE-induced Gaucher mouse models by assessing their body weight, motor behavior, and GCase activity. The study revealed various key findings regarding the protective role of NAG against the detrimental effects of CBE. Initially, all three experimental groups, including the control (without CBE), untreated and those treated with NAG, experienced increased body weight. However, a stark difference was observed after administering the 10th dose of CBE. The CBE injected mice without any NAG treatment exhibited a significant decrease in body weight, indicating severe adverse effects. In contrast, the mice receiving NAG treatment continued to gain weight, resembling the control group (Fig. 6a). The co-treatment of mice with the NAG might have counteracted the harmful effects of CBE, allowing the mice to grow similarly to the healthy control.

The motor behavior of the experimental groups was monitored using a wire-hanging test that assessed the ability of mice to cling to a hanging wire.^27^ The observation of the wire hanging test revealed that five doses of CBE injection had reduced motor scores in the untreated group when compared to the NAG treatment. Ten days of repeated administration of CBE had damaged all the motor abilities in the untreated group (without NAG) and fell immediately when placed over the wire, as shown in Fig. 6b and c (Videos S1–S6). On the other hand, NAG treatment showed normal motor behavior similar to the healthy control group. The findings indicate that NAG might act as a stabilizer, stabilizing the GCase enzyme, thereby preventing the degradation of GCase and helping maintain normal physiological activities in the Gaucher model.

Furthermore, the effect of NAG on the activity of GCase was evaluated using the 4MU-β-Glu substrate assay. The results of GCase activity revealed that NAG significantly enhanced the GCase activity of brain, liver, and spleen compared to the CBE-induced Gaucher mice and healthy control group (Fig. 6d). The findings from the above studies strongly suggest that NAG plays a vital role in stabilizing GCase and mitigating the harmful effects of CBE. Normal body weight and motor abilities indicate that NAG can cross the BBB and bind to the GCase within the neuronal tissues, which prevents Gaucher mouse models from the harmful effects of CBE, highlighting the therapeutic potential of NAG in GD conditions.

### 3.9. Preparation and characterization of IH + NAG

**Fig. 7:**
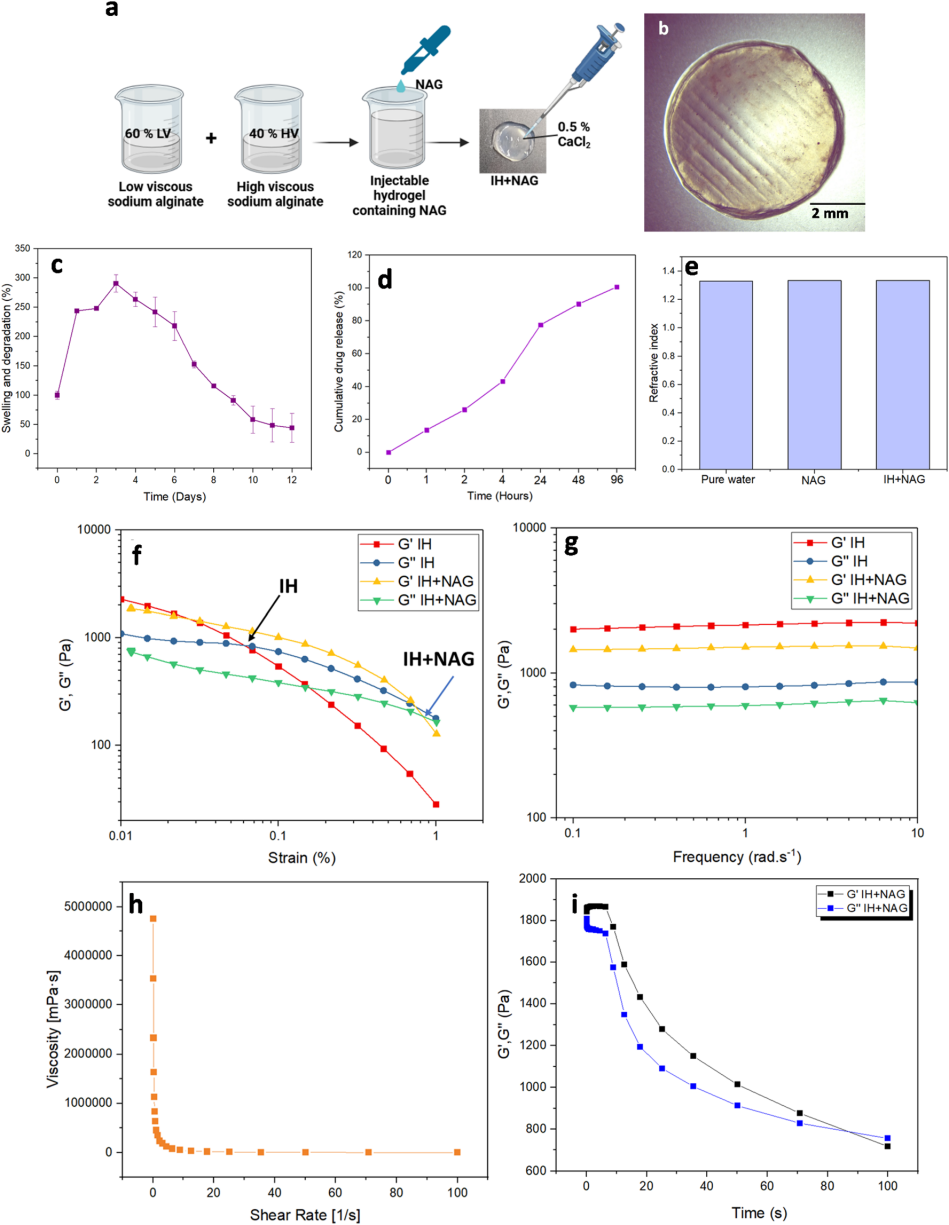
(a) Schematic representation showing the synthesis of injectable hydrogel containing NAG (IH + NAG), (b) microscopic image of IH + NAG captured using stereomicroscope, (c) swelling and degradation percentage of IH + NAG over time (*n* = 3), (d) the graph shows the slow and steady release of NAG from IH + NAG (e) refractive index of NAG and IH + NAG, (f) amplitude sweep at a constant frequency of 1 rad s^−1^, (g) frequency sweep at a constant shear strain of 1%, (h) the viscosity–shear rate curve of IH + NAG, (i) gelation kinetic of IH + NAG.

To ensure the slow and sustained release of NAG, an injectable alginate-containing NAG cocktail was developed that was crosslinked with CaCl_2_ solution to form an IH + NAG patch (Fig. 7a and b). A stability study using IH + NAG was performed at pH 7.4. The solidified IH + NAG maintained its initial shape till day 6 (Fig. S3) and retained its integrity for 12 days, corresponding to the therapeutic window's total duration. Further, a weight-based swelling study was performed that demonstrated an early increase in the weight of the hydrogel till day 3, followed by a slow and sustained decrease due to slow degradation of the hydrogel (Fig. 7c). The slow and sustained degradation of the IH + NAG facilitates the gradual release of the stabilizer from the hydrogel and facilitates GCase stability and activity.

A weight-based release study of the NAG from the injectable hydrogel indicates that NAG is released gradually from IH + NAG over the extended period of time (Fig. 7d). This slow and continuous release indicates IH + NAG prevents NAG from being released at once, maintaining a sustainable supply of NAG for a longer duration, and providing a prolonged therapeutic effect. Purity studies were determined by measuring the refractive index of NAG and IH + NAG in comparison to water, which was found to be comparable, indicating high purity of the hydrogel (Fig. 7e).^48^

The viscoelastic properties of IH and IH + NAG were evaluated by determining the compounds' yield stress and linear viscoelastic range (LVR) (Fig. 6f). In the LVR, both the storage modulus (*G*′) and the loss modulus (*G*′′) remain unaffected by applied shear strain, and hence, the curve of *G*′ is utilized to identify the limiting value in terms of strain as a percentage. After this range, the material starts to break. The limiting value was studied at the point where the *G*′ suddenly starts decreasing. A significant change in *G*′ for IH was observed at nearly 0.03% strain, whereas this plateau region was seen to increase in IH + NAG, indicating enhanced structural integrity and resistance to deformation. Additionally, IH was found to be quite fragile and starts to break at less than 0.1% of strain (indicated with a black arrow), whereas IH + NAG (marked with a blue arrow) could withstand strain even more than 0.1%. Both IH and IH + NAG were found to break with increasing strain, indicating their injectable and soft texture. Moreover, a frequency sweep in the LVR region was also conducted to study the structure and viscoelastic properties of the hydrogels (Fig. 7g). By applying a constant strain of 1% while varying the frequency, IH and IH + NAG consistently showed higher *G*′ than *G*′′ from 0.1 to 10 rad s^−1^, indicating elastic behavior. Additionally, the near constancy of both *G*′ and *G*′′ across the frequency range points to a stable network within the hydrogels. These findings demonstrate that adding NAG enhances the structural stability and resilience of the injectable hydrogels while maintaining their soft texture, making them suitable for biomedical applications.

It has been reported that viscosity decreases as the shear rate increases. Rheological analysis was performed to identify the effect of the accelerator nature on the rheological parameters and pseudo-plastic behavior of the hydrogel.^49^ A reduction in viscosity with the increase in shear rate confirms the non-Newtonian pseudo-plastic behavior of IH + NAG (Fig. 7h). Gelation kinetics of the IH + NAG were studied by oscillating a shear force during the cross-linking process. Storage modulus, *G*′, and loss modulus, *G*′′, was observed at 25 °C. The cross-over point between *G*′ and *G*′′ (*G*′ > *G*′′) also known as the gelation time and was found to be 80 s (Fig. 7i) for IH + NAG.^39^

### 3.10. A single dose of IH + NAG alleviates GD symptoms in CBE-induced Gaucher mouse model

**Fig. 8:**
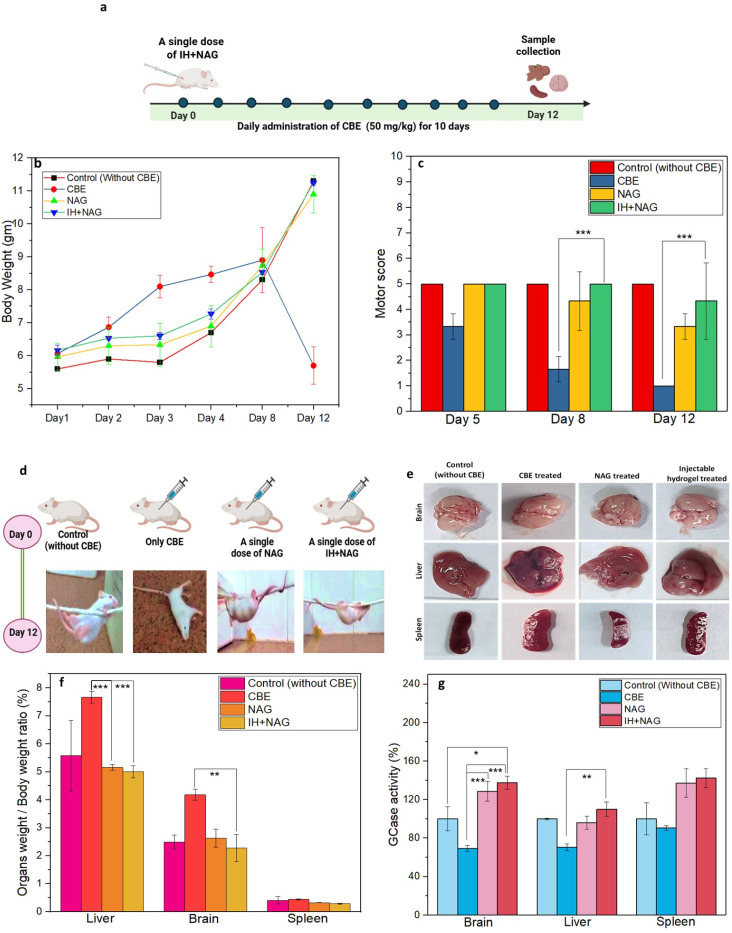
GCase stabilizing effects of IH + NAG in CBE-induced Gaucher mouse model: (a) scheme showing subcutaneous administration of a single dose of IH + NAG followed by continuous IP injections of CBE in 15 days mouse, (b) graph showing the weight of experimental group over time (c) motor score obtained by experimental groups during wire hanging test, (d) pictures of mice captured during wire hanging test, (e) images of organs isolated after 12 days of treatment, (f) organ weight of mice, (g) GCase activity of the untreated and treated group. A significant difference was observed in motor score, organ weight, and GCase activity among the NAG, IH + NAG, and control groups (*n* = 3, *p < 0.05).

To develop a single administration treatment, IH + NAG was developed and tested in the CBE-induced Gaucher mouse model (Fig. 8a). The hydrogel formulation consisted of alginate polymer loaded with NAG and was cross-linked using calcium chloride (CaCl_2_). Both sodium alginate and NAG consist of free hydroxyl groups which can be cross-linked using cationic cross-linker (Ca^2+^), stimulating the gelation of NAG. Thus, the Ca^2+^ ions present in calcium chloride enables linking of NAG and alginate molecules to themselves and to each other within the injectable hydrogel matrix. Inside the human body, the subcutaneous fluid (site where the injectable hydrogel formulation is administered) consists of large number of monovalent cations like Na^+^, K^+^*etc.* which will replace the cross-linker Ca^2+^ in the hydrogel matrix, resulting in the release of NAG from the formulation. The gelation occurs in pH = 7.4 and at room temperature (20–25 °C). Moreover, in the aqueous environment of the subcutaneous fluid, the hydrogel absorbs water and swells up, facilitating the sustained diffusion of the unbound NAG (soluble in water), which does not participate in the cross-linking process (Scheme S1).^50^

The body weight of mice treated with NAG and IH + NAG demonstrated a gradual increase in body weight, similar to that of the healthy control group, suggesting their protective effects against the metabolic disruptions caused by CBE by stabilizing the GCase. However, the untreated diseased group showed a decrease in body weight after the 8th dose of CBE, indicating a potential disease condition (Fig. 8b). Moreover, wire hanging test results demonstrated that NAG and IH + NAG treated groups consistently maintained their motor abilities similar to the healthy control group, even after prolonged exposure to CBE, with the IH + NAG treated mice scoring higher in the motor assessment (Fig. 8c, d and Videos S7–S10). Whereas, mice from the untreated CBE-induced group have a rapid decrease in motor abilities and abnormalities in the hindlimb and forelimb from day 5 to day 12, indicating metabolic and neuropathic GD-like conditions due to the effects of CBE. This shows the potential protective effect of IH + NAG for the prevention and treatment of GD, likely due to their ability to facilitate the slow release of NAG and stabilize GCase.^41^

A significant increase in organ weight was observed in the CBE-induced disease group, which might be due to the accumulation of glucocerebroside as a result of GCase inhibition inside the organs compared to NAG and IH + NAG treated groups that had normal organ weights similar to the healthy control mice (Fig. 8e and f). These results further correlate with the findings of GCase activity in organs (Fig. 8g). Compared to the untreated diseased group, enhanced GCase activity was observed in the mice's brain, liver, and spleen tissues with the NAG and IH + NAG treatment. Moreover, IH + NAG treatment shows higher GCase activity than the NAG due to the slow and sustained release of NAG from the hydrogel, leading to effective stabilization of GCase and prevention of GD for a longer duration.

**Fig. 9:**
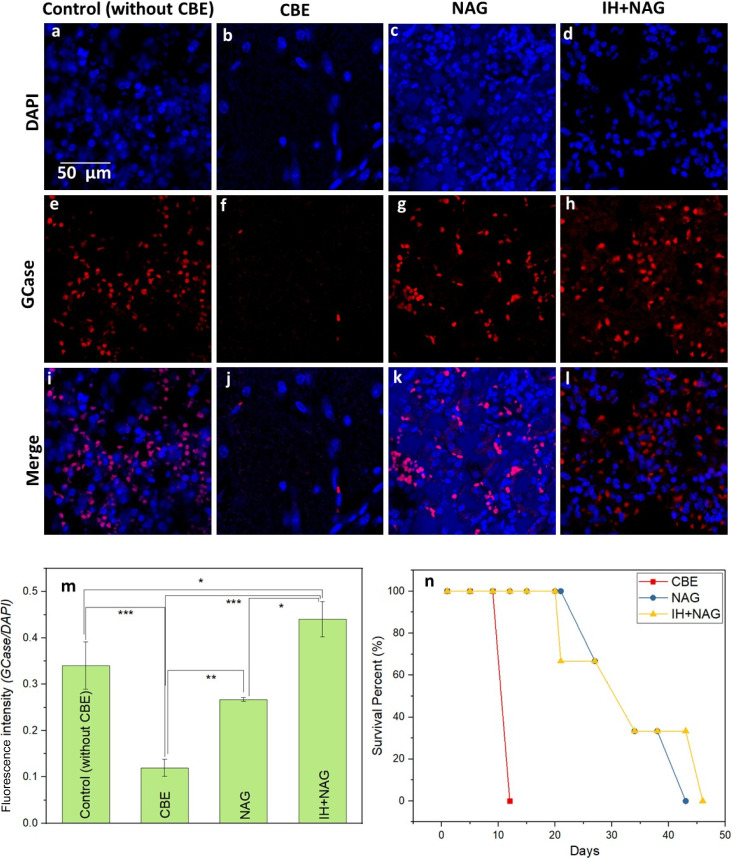
Immunostaining of brain tissues isolated from different experimental groups following 12 days of CBE administration in 15 days old mice, (a–d) slides stained with DAPI (e–h) slides stained with GCase antibody (i–l) merge slides (m) fluorescence intensity of GCase normalized with DAPI. (n) Graph showing survival study. A significant difference in the fluorescence intensity of anti-GCase was observed in NAG, IH + NAG, and control groups. (n) Survival study demonstrated that IH + NAG prolongs the survival rate of CBE induced Gaucher mouse model (*n* = 3, *p < 0.05).

Immunostaining was performed to visualize the presence of GCase enzyme in the isolated organs, particularly the brain, liver, and spleen. A high distribution of GCase enzymes was observed in the brain of NAG and IH + NAG treated groups similar to the control, whereas a notable reduction in GCase was observed in the brain, liver and spleen tissues of the untreated experimental group induced by CBE (Fig. 9a–l and S4, S5). This observation might be due to the stabilization effect of NAG, that binds to the GCase and help in its localization within the cells, resulting in increased GCase levels. Quantification analysis confirmed the higher distribution of GCase in the tissues of NAG and IH + NAG treated mice as compared to the untreated CBE group (Fig. 9m).

Further, the survival studies demonstrated significant differences among the experimental groups. All the mice in the untreated diseased group experienced fatalities after receiving the 12th dose of the CBE. In contrast, after treatment, the NAG and IH + NAG groups showed a remarkably longer survival duration, lasting for 43 and 46 days, respectively as shown in Fig. 8n. This supports the utility of the NAG treatment in improving the life-span of GD mice.

## 4. Discussion

In this study, we have developed a simple, cost-effective, easy-to-make injectable hydrogel construct containing NAG, a GCase stabilizer for the protection and therapy of GD. We repurposed a wide range of small molecules that are widely explored for their protein stabilizing ability for treating various genetic disorders from the published literature, which had not been studied for GD, and employed a comprehensive process pipeline for the pre-clinical identification of new glucocerebrosidase stabilizers. Based on initial docking results, NAG and DCA showed maximum binding and minimum inhibition constant with GCase. NAG emerged as the lead GCase stabilizer based on its low toxicity and high efficiency to increase GCase activity in both gene knockdown and CBE-induced Gaucher model *in vitro* and *in vivo*. Moreover, the results obtained from molecular docking and AAMD simulations demonstrated that NAG enhances the structural stability and compactness of both wildtype and mutant GCase proteins after binding. Further, a single-dose injectable hydrogel (IH + NAG) was designed for the prevention and treatment of GD with prolonged survival. It has already been reported that NAG is not genotoxic even at a higher concentration of 898 mg per kg body weight in the rats.^51^

After the initial docking results, NAG and DCA were tested for their effect on GCase enzyme through an *in vitro* CBE induced GD cell model. We observed significant increase in GCase activity due to NAG treatment. However, at this stage, it was unclear whether this enhancement in GCase activity is due to the competitive binding of NAG with the GCase, merely preventing the inhibitory effect of CBE. To confirm the role of NAG as a stabilizer of GCase, we went on to assess the effect of NAG in siRNA-mediated *in vitro* knockdown model of GD. Pre-designed siRNA against the *GBA1* gene could significantly reduce the GCase activity in RAW 264.7 cells, whereas co-treatment with NAG increased the GCase activity in residual enzymes (nearly 50%) even in siRNA-transfected cells. This effect is very similar to earlier research with GCase stabilizers, like isofagomine (IFG) and ambroxol that could enhance the activity of wild-type GCase even in normal cells and mice respectively.^25,52^

Several research groups, including Dehoux *et al.* and Wang *et al.*, have identified small molecules for GCase by virtual screening techniques and studied their effects *in vitro*.^16,53^*N*-(*n*-Nonyl)deoxynojirimycin (NN-DNJ), miglustat, and IFG are immunosugars that have been investigated for the treatment of neuronopathic forms GD.^52,54–56^ These substances effectively prolong lifespan in GD models by raising GCase activity and protein levels in the brain and visceral tissues. Nevertheless, a significant drawback of these treatments is their need for daily administration, which significantly raises the expense and burden for patients. Our approach provides a more economical and patient-friendly option requiring a single dose to prevent and treat GD. Withers and team treated L444P and N370S transgenic mouse model independently using either IMX8 or norIMX8 sulfone daily *via* IP injection for 14 days at a dose of 10 mg kg^−1^ day^−1^ and observed improvement in GCase activity in in liver, spleen, and brain tissues compared to untreated mice.^57^ In another study conducted by Sun *et al.* in a neuronopathic GD mouse model, they found that giving 600 mg per kg per day isofagomine for 20 days increased its lifespan to 40 days.^58^ In contrast, a single subcutaneous injection of a 150 mg per kg dose of IH + NAG markedly raised GCase activity and prolonged survival beyond 45 days. This indicates that IH + NAG is a promising option for treatment with a substantially lower dose than the current practice. Various high-throughput screenings have been done to identify small molecules with greater potential in treating lysosomal storage disorders, but most of their findings are limited to *in vitro* conditions.^59^ Fluorinated chaperone–β-cyclodextrin enhanced GCase activity in patient fibroblasts, but the effects are yet to be justified by proper animal experiments.^60^ Paul *et al.* demonstrated that small molecule inhibitors of proteinaceous amyloids like EGCG or NQTrp can reduce amyloid-like aggregation of glucocerebroside lipids in GD.^61^ Since GCase deficiency in the diseased condition can contribute to a wide range of detrimental pathways, prevention of lipid accumulation seems to provide temporary relief in GD management. We targeted the root cause of the disease, and our lead small molecule successfully restored the GCase activity in cellular and mouse models, along with improving pathological symptoms (motor activity).

A significant innovation of this study is using an injectable hydrogel as a delivery system for NAG for the prevention and treatment of GD. Developing an injectable hydrogel-based single dose of GCase stabilizer represents a substantial advancement, as to our knowledge, no prior study has explored this type of strategy. While this study demonstrates significant progress, it acknowledges certain limitations. The effects of IH + NAG are evaluated only in CBE-induced models, and the detrimental effects of CBE slowly reverse once its administration is stopped. Future research will evaluate the effects of IH + NAG in GD patient-derived cells and mutant animal models to further refine our promising therapeutic strategy. Additionally, we will focus on assessing long-term toxicity and therapeutic studies of IH + NAG in GD and other lysosomal storage disorders.

## 5. Conclusion

A drug-repurposing strategy was employed for virtual screening of 30 different small molecules, which led to the identification of NAG, that was found to be biocompatible and supported GCase stability and activity. A novel, cost-effective, patient-friendly IH + NAG construct containing NAG was developed to meet current therapies' challenges. These hydrogel constructs do not require surgery or multiple administrations. A single administration of IH + NAG enhanced the GCase activity *in vitro* and *in vivo*, providing protection and therapeutic potential for all forms of GD. Moreover, our novel construct can potentially treat other neurodegenerative diseases associated with a deficiency in the GCase enzyme.

## Conflicts of interest

The authors declare no conflicts of interest.

## Supplementary Material

RA-015-D5RA04610F-s001

## Data Availability

The data supporting this article have been included as part of the supplementary information (SI). Additional data will be made available upon request as reasonably possible. Supplementary information is available. See DOI: https://doi.org/10.1039/d5ra04610f.
