# Supplementary material for: An injectable hydrogel containing N-acetylglycine for the treatment of Gaucher disease
Source: RSC Adv. 2025 Oct 28;15(48):41220–40. doi: 10.1039/d5ra04610f (PMC12560097; doi:10.1039/d5ra04610f)
Supplement: RA-015-D5RA04610F-s001 [file RA-015-D5RA04610F-s001.pdf]

Supplementary Information (SI) for RSC Advances.
This journal is © The Royal Society of Chemistry 2025

Supporting Information

An injectable hydrogel containing N-acetylglycine for the treatment of Gaucher disease

Lipi Pradhan,[a#] Sumit Manna,[a#] Amar Jeet Yadav,[b] Bajrang Bajrang,[a] Debayani Chakraborty,[a] Khushboo Bhagat,[b] Shikha Tripathi,[c] Avanish Singh Parmar,[c] Aditya K. Padhi,[b] Sudip Mukherjee*[a]*

[a]School of Biomedical Engineering, IIT (BHU), Varanasi,221005, UP, India

[b]Laboratory for Computational Biology & Biomolecular Design, School of Biochemical Engineering, IIT (BHU), Varanasi,221005, UP, India

[c]Department of Physics, IIT (BHU), Varanasi,221005, UP, India

[#]Equal contributing first author

**Corresponding author: Dr. Aditya K. Padhi, aditya.bce@iitbhu.ac.in*

**Corresponding and communicating author: Dr. Sudip Mukherjee, sudip.bme@iitbhu.ac.in, Phone Number: +91-7980659213*

1. Methods and Materials

1.1. Molecular docking analysis of WT and mutant GCase with NAG

Detailed molecular docking and simulations were conducted using AutoDock4 to examine NAG's binding modes, binding affinities, and complex stability with both WT-GCase and mutant-GCase.Before docking, water molecules and other counter ions were removed from the protein structure. Polar hydrogen atoms were then added to ensure amino acids' correct ionization and tautomeric states, followed by merging non-polar hydrogens. Kollman united atom charges and solvation parameters were assigned, and the final structures were saved in PDBQT format. Gasteiger charges were assigned to the ligand (NAG), and docking experiments were performed using the Lamarckian Genetic Algorithm with a population size of 150 and 2.5 million energy evaluations. Default settings were applied for parameters such as crossover and mutation rates. Blind docking was carried out with grid boxes sized to fully encompass the protein, measuring 126×106×126 Å for both WT and mutant GCase, centered on the macromolecule with a default grid point spacing of 0.575 Å. Pre-generated grid maps, capturing interaction energy grids based on ligand atom probes interacting with the receptor target, were obtained using AutoGrid. The results were then clustered into groups of similar

conformations based on root mean square deviation (RMSD) and orientation. Each docking experiment produced multiple binding conformations, from which the most favorable models were selected based on docking scores and the number of interactions formed. The docking score, indicative of the predicted binding affinity between the NAG and the GCase, was a critical evaluation metric, with lower scores representing stronger binding. The docked complexes were analyzed in detail to identify residue-level interactions and compare binding differences between the WT-GCase and mutant-GCase.

1.2. Analysis of molecular interactions in GCase-NAG docked complexes

The protein-ligand interactions within the GCase-NAG docked complexes were analyzed to identify the key amino acid residues of GCase involved in NAG binding. Furthermore, three-dimensional visualization of the wild-type and mutant GCase-NAG complexes was performed using PyMOL to provide a comprehensive structural understanding of these interactions.

1.3. Analysis of physicochemical properties and free energy landscape from AAMD simulation trajectories

In AAMD simulations, water was used as the explicit solvent. Specifically, we employed the SPC/E (Extended Simple Point Charge) water model, which is widely used for biomolecular systems due to its balance between computational efficiency and accuracy in reproducing the properties of liquid water. Each system was fully solvated in a cubic box with a minimum 1.0 nm buffer from the solute to the box edges to ensure proper solvation and avoid artefacts due to periodic boundaries. Next, the physicochemical properties and molecular interactions were analyzed using GROMACS v.22 toolsets. Key metrics such as root-mean-square deviation (RMSD), root-mean-square fluctuations (RMSF), radius of gyration (Rg), and hydrogen bond interactions were computed using gmx_mpi rms, gmx_mpi rmsf, gmx_mpi gyrate, and gmx_mpi hbond modules, respectively, based on the AAMD simulation trajectories. Principal component analysis (PCA) was performed to further probe system dynamics to identify dominant motion modes and collective movements within the systems. This involved constructing a covariance matrix and deriving eigenvectors and eigenvalues with gmx_mpi covar and gmx_mpi anaeig modules.[1] The free energy landscape (FEL) of the WT and mutant GCase, WT-GCase-NAG, and mutant-GCase-NAG complex models was also mapped using the gmx_mpi sham module. Three-dimensional contour plots were generated to visualize energy variations and assess stability across distinct conformational states.

1.4. End-state binding free energy calculations for GCase-NAG Complexes

The end-state binding free energies between WT and mutant GCase with NAG were evaluated using MM/PBSA approach, performed through the gmx_MMPBSA tool, which integrates AMBER's MMPBSA.py script. This analysis was conducted within a Python 3 Miniconda environment with all necessary gmx_MMPBSA dependencies.[2-3] Trajectory, topology, and index files were prepared for the analysis, with GCase and NAG defined as separate groups. Input files included GROMACS formats such as portable topology files (.itp), structural files (.gro, .pdb, or .psf), trajectory files (.xtc), and run topology files (.tpr). The binding free energy calculations utilized equilibrated and stable AAMD trajectory data, ensuring reliability and representation of the simulation's steady-state behavior. These calculations provided critical insights into the energetic contributions of GCase residues and NAG atoms, shedding light on the molecular interactions that drive the stability and binding affinity of the GCase-NAG complex.

1.5. Cell viability assay:

A colorimetric MTT assay was performed to study the effects of the top 5 drugs on the viability of cells. The MTT assay is based on the ability of metabolically active cells to transform a water-soluble dye[3-(4,5-dimethylthiazol-2-yl)-2,5-diphenyltetrazolium bromide] into an insoluble formazan by mitochondrial succinate dehydrogenase. When cells die, they lose their ability to convert MTT into formazan; thus, color formation presumably serves as a marker of the viable cells.[4] Quantitative determination of the amount of formazan estimates the number of live cells. We examined the effects of five lead 5 drugs on the viability of HEK-293 (human embryonic kidney 293 cell line) cells. 1×10^4 HEK-293 cells were seeded in each well of a 96-well plate supplemented with 100 µL DMEM media. The cells were allowed to grow for 24 hours. After the incubation period, the cells were exposed to a higher (5000 µM) and a lower (25 µM) concentration of drugs, followed by another 24 hours of incubation at 37 °C. The DMEM media was replaced by 100 µL (0.5 mg/mL) MTT suspension. The plates were placed in the dark at 37 °C and 5% CO_2 for 4 hours. After the incubation period, the DMEM media was discarded, and 100 µL of DMSO: MeOH (1:1) was added to dissolve the formazan crystal. The absorbance of the mixture was analyzed using a multi-plate reader at 570 nm. After that, the cell viability (%) was calculated using the following formula: % cell viability=(As/Ac) ×100

As and Ac denotes absorbance of sample and control at 570 nm.

1.6. Chorioallantoic membrane (CAM) assay:

CAM is an extraembryonic layer of the developing chicken embryo containing dense capillaries and blood vessels, and acts as an organ for gaseous exchange.[5] It is widely used as a temporary host for grafted substances and biomaterials to study their biocompatibility and safety in living systems.[6] We examined the biocompatibility of the top 5 leads utilizing the CAM assay. Initially, 1-day-old fertilized eggs were purchased from a poultry farm and placed inside an incubator for the next 4 days, providing proper temperature and humidity. On day 5, a small window was created over the eggshell to visualize the developing embryo. A 6 mm Whatman filter paper disc soaked with a lead drug (5 mM) was then placed carefully over the CAM layer. The window was then sealed using parafilm and again incubated for an additional 6 hours. The change in developing blood vessels was monitored by capturing images at various time points with the help of a Magnus MagZoom TZM6 Trinocular Stereo Zoom Microscope. The images obtained were later processed using FijiJ and Angiotool software. The fold changes in various parameters were analyzed using the microscopic images obtained at 0 and 6 hours.

1.7. Motor behavior:

Supporting Table 1. The conditions and scores are summarized below

Sl. No	Condition	Score
1	The mouse fell from the hanging wire the moment it was set in place.	0
2	Mouse tries to cling to the hanging wire	0.5
3	Mouse managed to hold the wire for at least ten seconds	1
4	Using two forepaws, the mouse held the hanging wire for over ten seconds	2
5	Mouse clung on the hanging wire for over ten seconds using 3-4 paws	3
6	Mouse managed to hold the hanging wire using all 4 paws and its tail for over ten seconds	4
7	The mouse starts moving actively on the hanging wire	5

1.8. Stability study of IH+NAG:

To study the stability of our injectable hydrogel formulation, 100 µl of alginate-NAG solution was loaded onto circular molds, adding an equal volume of cross-linking solution (0.5 % $CaCl_2$). The resultant mixture was allowed to solidify, and the prepared hydrogel-NAG patches were immersed in a pH 7.4 solution. Microscopic images ((Magnus MagZoom TZM6 Trinocular Stereo Zoom Microscope) of the solidified hydrogels were captured to assess the shape and integrity of the hydrogel-NAG patches using a stereomicroscope starting from the day of preparation, day 0, to day 12. The weight of the solidified hydrogel-NAG formulation was also measured daily during this period.

1.9. In vitro release study of NAG from IH+NAG:

A total of 150 mg of N-acetylglycine (NAG) was combined with 2 ml of alginate solution and poured into a well of a 24-well plate. Subsequently, 2 ml of the crosslinking solution (0.5% $CaCl_2$) was added to the well and allowed to incubate for 10 minutes to facilitate the crosslinking process, forming the IH+NAG patch. The IH+NAG patch was then incubated in the crosslinking solution, and at various time intervals, samples of the crosslinking solution were collected in an Eppendorf tube and replaced with 2 ml of fresh crosslinking bath. The weight of the solution at each time point was recorded and subtracted from the initial measurement. The cumulative weight of the NAG released into the solution was calculated and plotted as a percentage of cumulative drug release.

2. Results and Discussion:

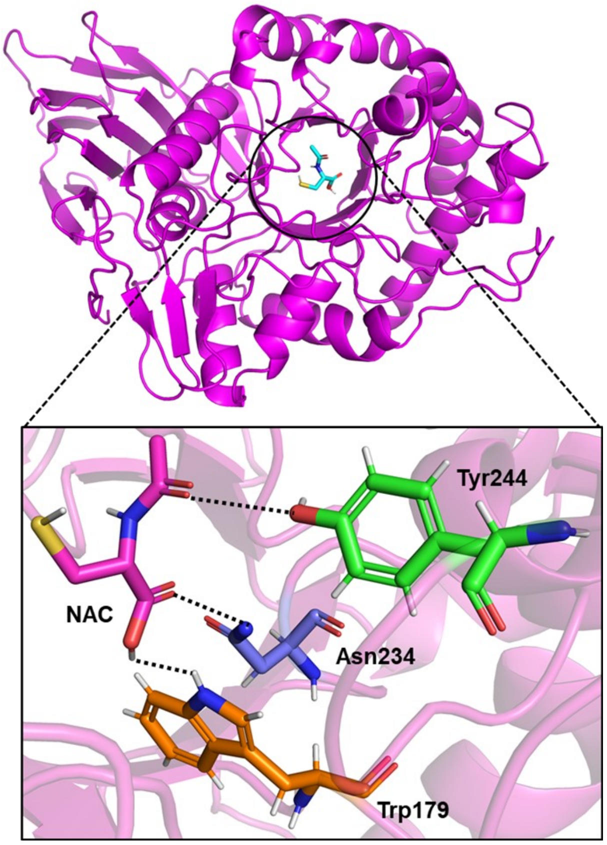

Supporting Figure 1. 3D visualization of the docked complex of human GCase with N-acetylcysteine (NAC). The key interacting recidues are labelled and represented as stick models. NAC is bound to GCase with hydrogen bonds illustrated by black dotted lines.

Supporting Table 2. The table summarizes the systems analyzed for all-atom molecular dynamics simulations and their respective simulation durations.

Sl. No	System under study	Simulation time
1.	WT-GCase	100 ns
2.	Mutant-GCase	100 ns
3.	WT-GCase-NAG	100 ns
4.	Mutant-GCase-NAG	100 ns

2.1. Principal component analysis and free energy landscape of GCase and GCase-NAG models from AAMD simulations

Principal component analysis (PCA) and free energy landscape profiling helped to understand the overall motions and structural dynamics of WT and mutant GCase and their NAG-bound complexes. PCA was employed to analyze collective atomic motions, focusing on eigenvectors with the largest eigenvalues to capture the dominant dynamic subspace. Stable PCA clusters were identified for WT-GCase-NAG, mutant-GCase-NAG, WT-GCase, and mutant-GCase by evaluating the backbone atoms during equilibrated phases of the simulations. The compactness of these complexes was further confirmed through trace values of covariance matrices, with WT-GCase-NAG and mutant-GCase-NAG exhibiting values of 7.60 and 9.12 nm^2, respectively, compared to 18.47 nm^2 and 29.42 nm^2 for WT-GCase and mutant-GCase. The dominant eigenvectors revealed that the first two accounted for the majority of the system dynamics, offering valuable insights into structural correlations. Gibbs free energy landscapes (FEL) generated from the first two principal components (PC1 and PC2) highlighted that the WT-GCase-NAG complex adopts more stable and compact conformations compared to its mutant counterpart and the unbound GCase models. This stability is evident in the FEL plots, where WT-GCase-NAG features more extensive deep blue low-energy basins, signifying stable low-energy states. At the same time, the mutant-GCase-NAG and other systems exhibit higher energy states (indicated by red regions) (**Figure 3. f-i**) These results confirm distinct structural and dynamic behaviors across the four systems, aligning with observations from AAMD simulations and providing critical insights into the stability and conformational preferences of WT and mutant GCase models.

Supporting Table 3. The table presents the binding free energy values and various energy contributions (in kcal/mol) for interactions between WT and mutant GCase with NAG. These values were computed using the MM/PBSA approach, based on stable trajectories from MD simulations of the WT-GCase-NAG and mutant-GCase-NAG complexes.

Molecular systems	Energy component	Average	SD $(Prop.)^a$	$(SD)^b$	SEM $(Prop.)^c$	$(SEM)^d$
WT-GCase-NAG	ΔBOND	0	1.27	0	0.02	0
	ΔANGLE	0	2.71	0	0.04	0
	ΔDIHED	0	1.17	0	0.02	0
	ΔVDWAALS	-13.68	0.32	2.66	0	0.04
	ΔEEL	-41.41	8.62	6.92	0.12	0.1
	Δ1-4 VDW	0	2.17	0	0.03	0
	Δ1-4 EEL	0	2.07	0	0.03	0
	ΔEGB	38.93	1.11	4.48	0.02	0.06
	ΔESURF	-2.85	0.02	0.12	0	0
	ΔGGAS	-55.1	8.62	6.39	0.12	0.09
	ΔGSOLV	36.09	1.11	4.46	0.02	0.06
	**ΔTOTAL**	**-19.01**	**8.7**	**3.2**	**0.12**	**0.05**
Mutant-GCase-NAG	ΔBOND	0	1.35	0	0.02	0
	ΔANGLE	0	2.67	0	0.04	0
	ΔDIHED	0	1.24	0	0.02	0
	ΔVDWAALS	-15.1	0.41	2.33	0.01	0.03
	ΔEEL	-10.2	4.38	4.77	0.06	0.07
	Δ1-4 VDW	0	2.13	0	0.03	0
	Δ1-4 EEL	0	1.77	0	0.02	0
	ΔEGB	15.2	0.28	3.61	0	0.05
	ΔESURF	-2.32	0.02	0.34	0	0
	ΔGGAS	-25.3	4.4	5.81	0.06	0.08
	ΔGSOLV	12.88	0.28	3.51	0	0.05
	**ΔTOTAL**	**-12.42**	**4.41**	**3.65**	**0.06**	**0.05**

[a] The standard deviation (SD) is acquired through the propagation of the uncertainty formula. [b] SD represents the sample standard deviation. [c] The standard error of the mean (SEM) is determined through the propagation of the uncertainty formula. [d] SEM denotes the sample standard error of the mean.

Supporting Table 4. The table presents a detailed decomposition analysis of various energies and overall binding energy of the complexes derived from the equilibrated and stable phase of AAMD simulations. All energy values are reported in kcal/mol.

[a] The standard deviation (SD) is acquired through the propagation of the uncertainty formula. [b] SD represents the sample standard deviation. [c] The standard error of the mean (SEM) is

**Molecular systems**	**Complex energy component**	**Average**	**SD** $(Prop.)^a$	$(SD)^b$	**SEM** $(Prop.)^c$	$(SEM)^d$
WT-GCase-NAG	BOND	1450.98	31.17	31.17	0.44	0.44
	ANGLE	3784.02	45.56	45.56	0.65	0.65
	DIHED	-11967.9	35.88	35.88	0.51	0.51
	VDWAALS	-4266.52	29.43	29.43	0.42	0.42
	EEL	-31119.9	131.58	131.58	1.87	1.87
	1-4 VDW	2480.57	22.22	22.22	0.32	0.32
	1-4 EEL	11526.53	30.18	30.18	0.43	0.43
	EGB	-4520.01	101.97	101.97	1.45	1.45
	ESURF	137.93	2.04	2.04	0.03	0.03
	GGAS	-28112.2	154.66	117.39	2.2	1.67
	GSOLV	-4382.08	101.99	100.95	1.45	1.43
	**TOTAL**	**-32494.3**	**185.26**	**63.21**	**2.63**	**0.9**
Mutant-GCase-NAG	BOND	1452.55	30.78	30.78	0.44	0.44
	ANGLE	3786.38	46.06	46.06	0.65	0.65
	DIHED	-11980.3	35.38	35.38	0.5	0.5
	VDWAALS	-4169.84	29.25	29.25	0.41	0.41
	EEL	-30954.4	117.43	117.43	1.66	1.66
	1-4 VDW	2475.56	22.67	22.67	0.32	0.32
	1-4 EEL	11552.32	35.16	35.16	0.5	0.5
	EGB	-4647.08	97.42	97.42	1.38	1.38
	ESURF	145.78	2.5	2.5	0.04	0.04
	GGAS	-27837.7	143.93	120.37	2.04	1.7
	GSOLV	-4501.3	97.45	96.36	1.38	1.36
	**TOTAL**	**-32339**	**173.82**	**63.92**	**2.46**	**0.9**

determined through the propagation of the uncertainty formula. [d] SEM denotes the sample standard error of the mean.

Decomposition of energetic contributions highlighted that ΔVDWAALS, ΔEEL, ΔEGB, ΔGGAS, and ΔSOLV were the key factors driving the binding free energies of the GCase-NAG complexes (**Supporting table 4**).

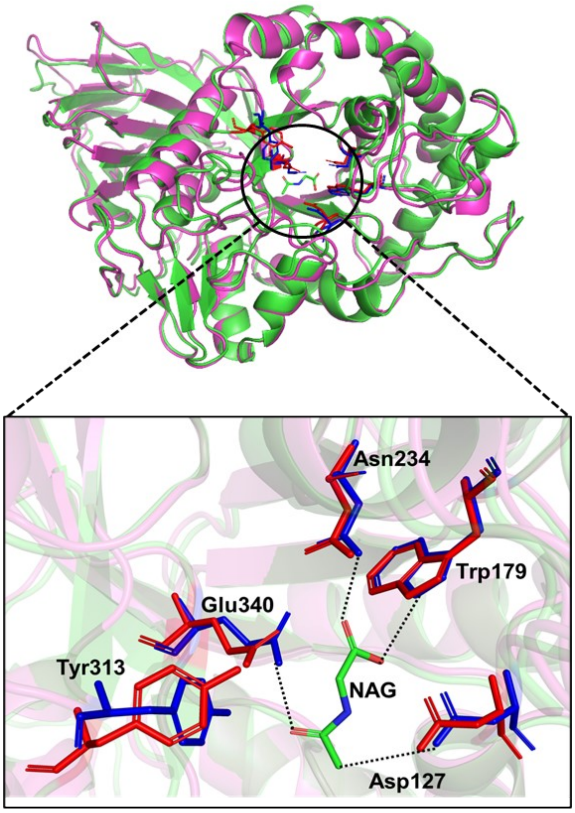

Supporting Figure 2. Close-up views of the active site region of WT-GCase (magenta) and mutant GCase (green). Figure shows close-up views of the active site region in WT-GCase (magenta) and mutant GCase (green). The key active-site residues, Asn127, Asn179, Asn234, and Glu340, are represented as stick models. In the WT-GCase, residues are highlighted in blue, while the corresponding residues in the N370S mutant GCase are shown in red. Structural superimposition reveals conformational and orientational changes in these residues induced by the mutation. N-acetylglycine (NAG) is bound to the WT-GCase, with hydrogen bonds illustrated by dashed lines. In contrast, NAG binds to a different site in the mutant GCase due to the mutation-induced conformational alterations in the orientation of key active-site residues.

2.2. Wire hanging test of 8 days old CBE-induced Gaucher mouse model after 5th and 10th dose of CBE injection

Supporting Video 1: Wire hanging test of healthy control (without CBE) on day 5

https://drive.google.com/file/d/18b9XLVZeUKGG2QRJn-iLzJVxuFe6sozP/view?usp=drive_link

Supporting Video 2: Wire hanging test of untreated CBE group on day 5 https://drive.google.com/file/d/1jJo4_MnJ1V9z7p6zn0qYHkniYTg0HVJM/view?usp=drive_link

Supporting Video 3: Wire hanging test of NAG treated (CBE+NAG) group on day 5 https://drive.google.com/file/d/1OqWER8JVsoPVP49v63D7dJfIBfGy2pll/view?usp=drive_link

Supporting Video 4: Wire hanging test of healthy control (without CBE) on day 10 https://drive.google.com/file/d/1vkAQZd5Rfadz7AtaShrEf9WU_TTPYUaz/view?usp=drive_link

Supporting Video 5: Wire hanging test of untreated CBE group on day 10

https://drive.google.com/file/d/1fB0g7dSlzPIuX-xYzqLLPJaX7VWghOxK/view?usp=drive_link

Supporting Video 6: Wire hanging test of NAG treated (CBE+NAG) group on day 10

https://drive.google.com/file/d/1aw53L077a_oLs_Ojb6gsfKYDjDq6EQ99/view?usp=drive_link

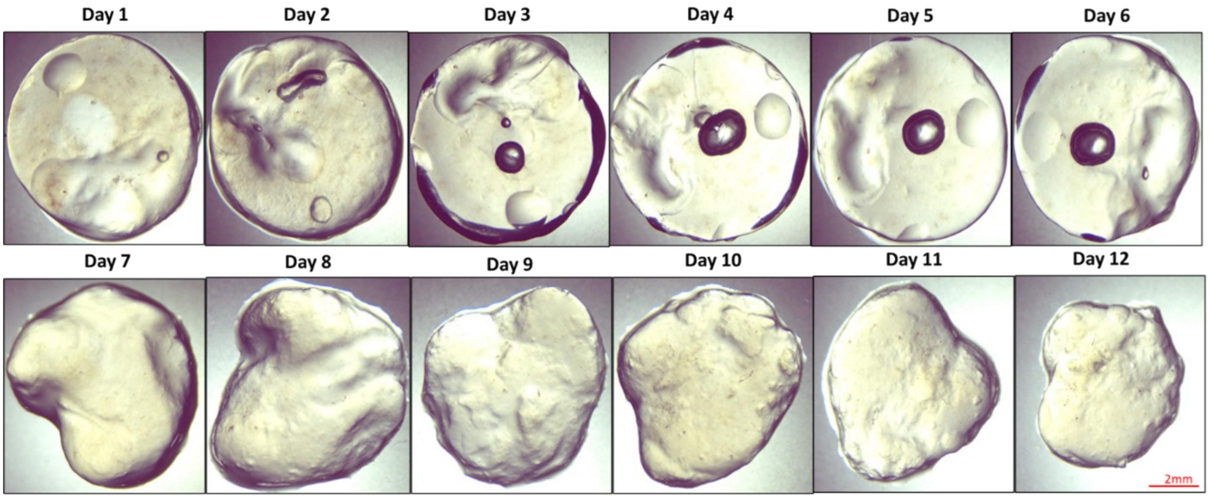

2.3. Preparation and characterization of IH+NAG

Supporting Figure 3. Stability study of IH+NAG in pH 7.4 over a period of 12 days

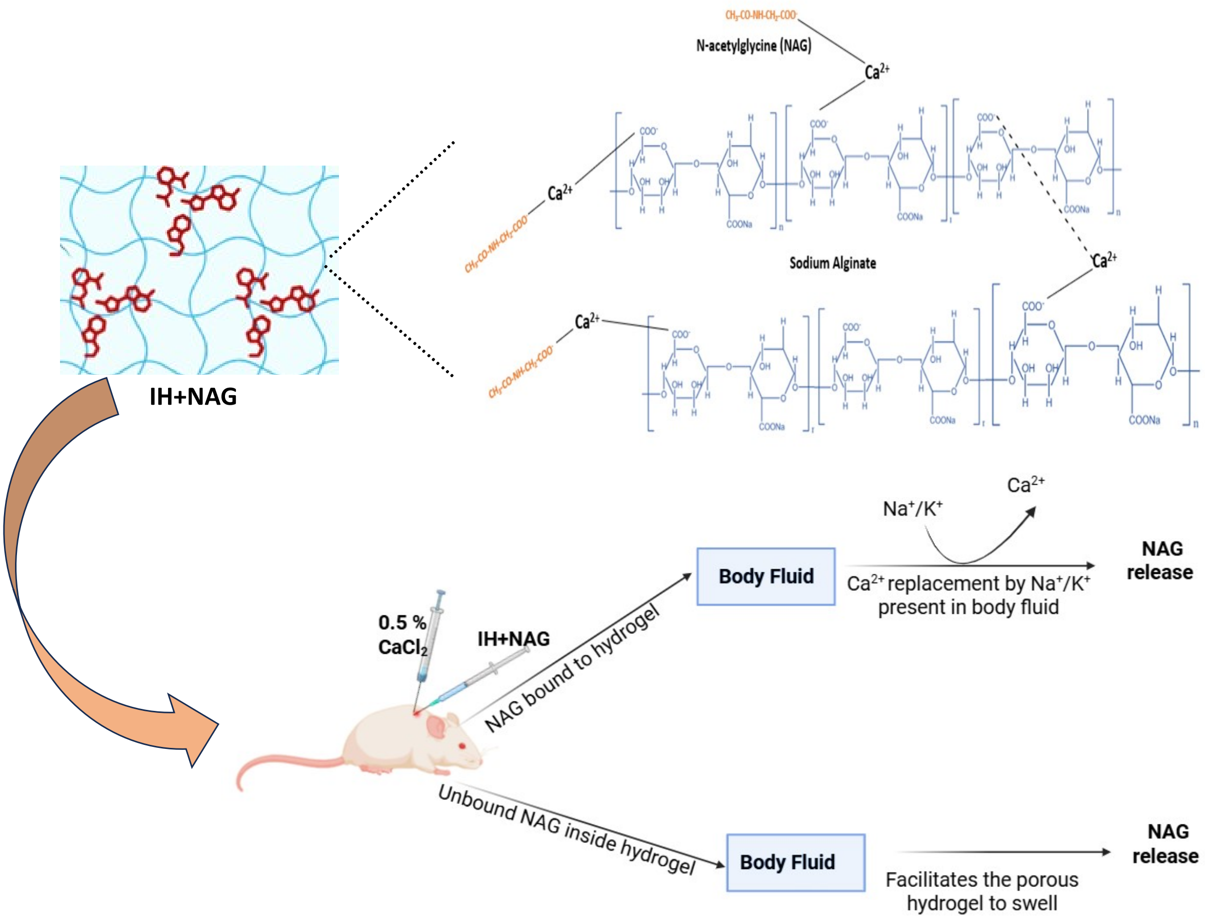

Supporting Scheme 1. Schematic representation showing the probable mechanism of gelation and NAG release from the hydrogel

2.4. Wire hanging test of 15 days old CBE-induced Gaucher mouse model after 12th dose of CBE injection

Supporting Video 7: Wire hanging test of healthy control (without CBE) on day 12

https://drive.google.com/file/d/1T_tUuKRb9xtFCMyfy0LDmgM88qi9b-HZ/view?usp=drive_link

Supporting Video 8: Wire hanging test of untreated CBE group on day 12

https://drive.google.com/file/d/14SNi4sQeucO0t_gCE-cD4WVZ3dSZe3jv/view?usp=drive_link

Supporting Video 9: Wire hanging test of NAG treated group on day 12 https://drive.google.com/file/d/10QCaF4J380Yz2y7ODFIeV45EjAwxT585/view?usp=drive_link

Supporting Video 10: Wire hanging test of IH+NAG treated group on day 12 https://drive.google.com/file/d/1wgFFZHYvxM6FtQfbh2cuNZhiq9U3C62e/view?usp=drive_link

2.5. Immunostaining of liver and spleen tissues isolated from experimental groups following 12 days of CBE administration.

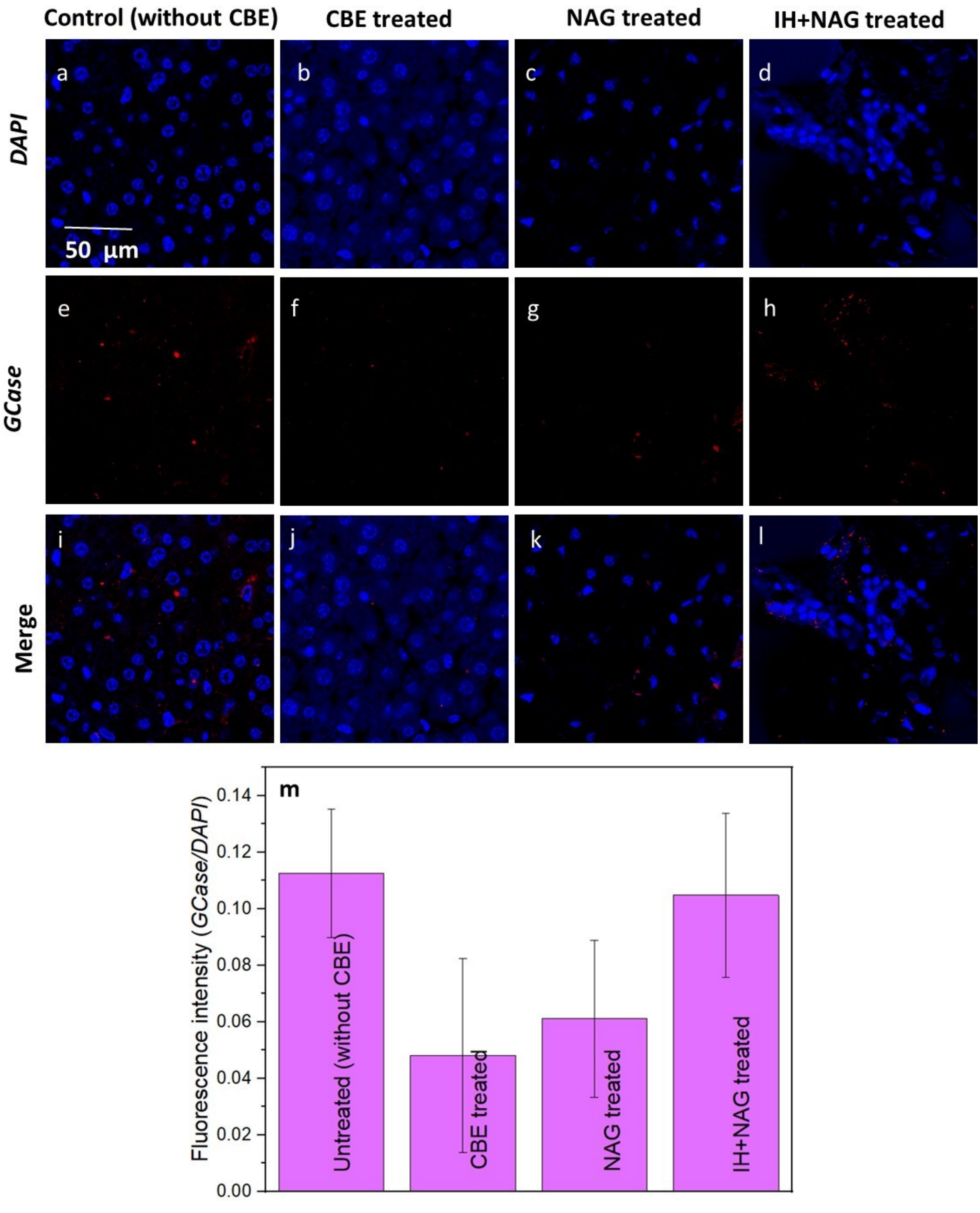

Supporting Figure 4. Immunostaining of liver tissue sections obtained from experimental groups following 12 days of CBE administration in 15 days old mice (a-d) slides stained with DAPI (e-h) Slides stained with GCase antibody (i-l) merge slides (m) fluorescence intensity of GCase normalized with *DAPI*.

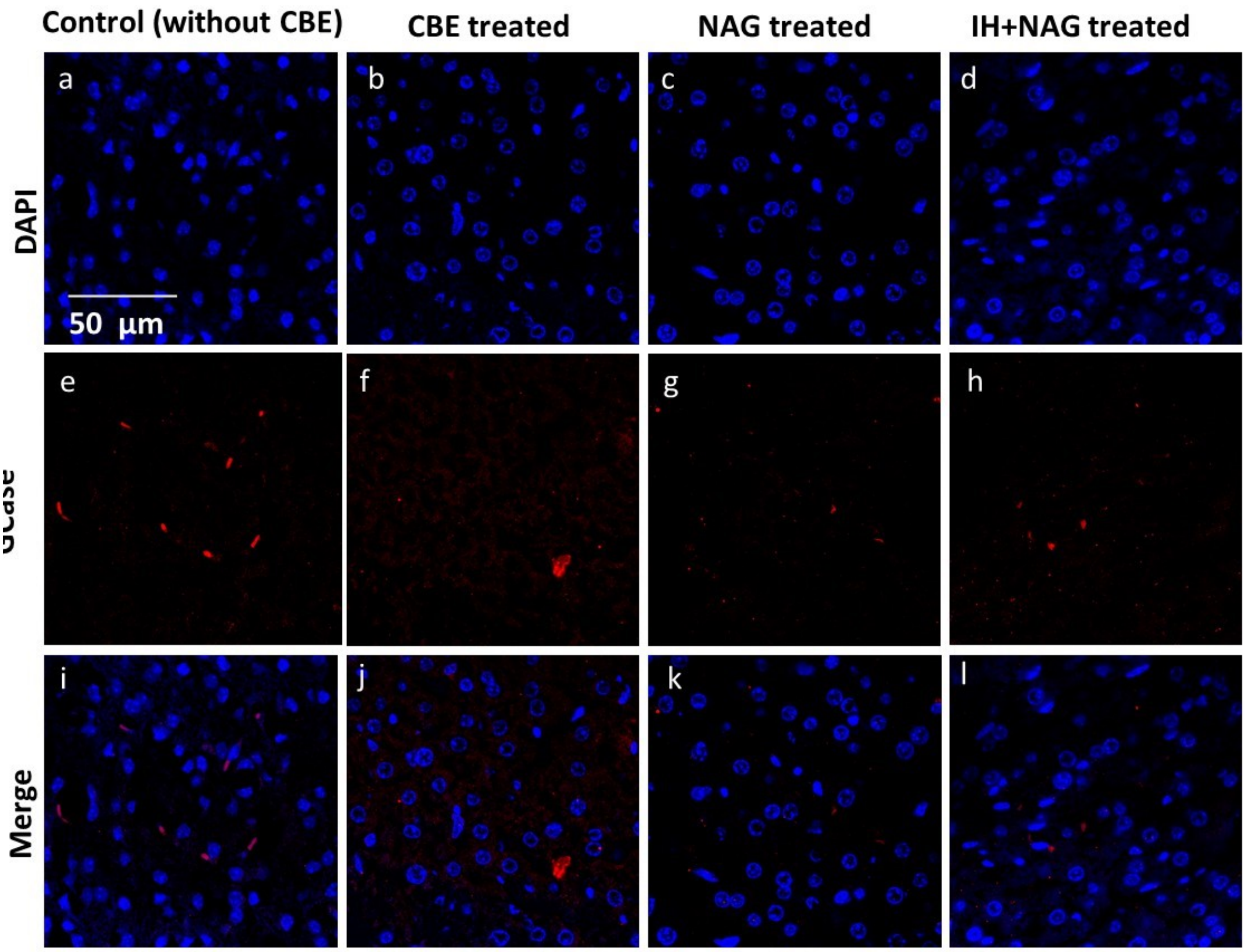

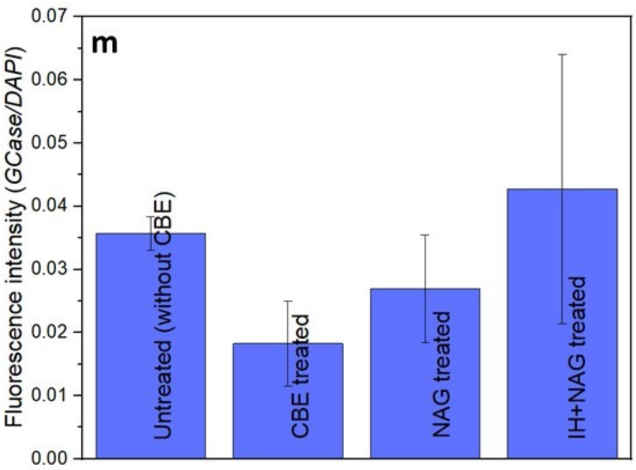

Supporting Figure 5. Immunostaining of spleen tissue sections obtained from experimental groups following 12 days of CBE administration in 15 days old mice(a-d) slides stained with DAPI (e-h) Slides stained with GCase antibody (i-l) merge slides (m) fluorescence intensity of GCase normalized with *DAPI*.

References

1. Greenacre, M.; Groenen, P. J.; Hastie, T.; d'Enza, A. I.; Markos, A.; Tuzhilina, E., Principal component analysis. *Nature Reviews Methods Primers* **2022,** *2* (1), 100.
2. Valdés-Tresanco, M. S.; Valdés-Tresanco, M. E.; Valiente, P. A.; Moreno, E., gmx_MMPBSA: a new tool to perform end-state free energy calculations with GROMACS. *Journal of chemical theory and computation* **2021,** *17* (10), 6281-6291.
3. Miller III, B. R.; McGee Jr, T. D.; Swails, J. M.; Homeyer, N.; Gohlke, H.; Roitberg, A. E., MMPBSA. py: an efficient program for end-state free energy calculations. *Journal of chemical theory and computation* **2012,** *8* (9), 3314-3321.
4. Gutiérrez, L.; Stepien, G.; Pérez-Hernández, M.; Pardo, J.; Grazú, V., Nanotechnology in drug discovery and development. **2017**.
5. Mangir, N. i.; Dikici, S.; Claeyssens, F.; MacNeil, S., Using ex ovo chick chorioallantoic membrane (CAM) assay to evaluate the biocompatibility and angiogenic response to biomaterials. *ACS Biomaterials Science & Engineering* **2019,** *5* (7), 3190-3200.
6. Butler, K. S.; Brinker, C. J.; Leong, H. S., Bridging the in vitro to in vivo gap: using the chick embryo model to accelerate nanoparticle validation and qualification for in vivo studies. *ACS nano* **2022,** *16* (12), 19626-19650.
